# Systematic review of studies generating individual participant data on the efficacy of drugs for treating soil-transmitted helminthiases and the case for data-sharing

**DOI:** 10.1371/journal.pntd.0006053

**Published:** 2017-10-31

**Authors:** Julia B. Halder, Joanne Benton, Amélie M. Julé, Phillipe J. Guérin, Piero L. Olliaro, María-Gloria Basáñez, Martin Walker

**Affiliations:** 1 Department of Infectious Disease Epidemiology and London Centre for Neglected Tropical Disease Research, Imperial College London, Norfolk Place, London, United Kingdom; 2 Independent Researcher, London, United Kingdom; 3 Infectious Diseases Data Observatory (IDDO), University of Oxford, Oxford, United Kingdom; 4 Centre for Global Health and Tropical Medicine, Nuffield Department of Medicine, University of Oxford, Oxford, United Kingdom; 5 World Health Organization Special Programme on Research and Training in Tropical Diseases (TDR), Geneva, Switzerland; 6 Department of Pathobiology and Population Sciences and London Centre for Neglected Tropical Disease Research, Royal Veterinary College, Hatfield, United Kingdom; University of Pennsylvania, UNITED STATES

## Abstract

**Background:**

Preventive chemotherapy and transmission control (PCT) by mass drug administration is the cornerstone of the World Health Organization (WHO)’s policy to control soil-transmitted helminthiases (STHs) caused by *Ascaris lumbricoides* (roundworm), *Trichuris trichiura* (whipworm) and hookworm species (*Necator americanus* and *Ancylostama duodenale*) which affect over 1 billion people globally. Despite consensus that drug efficacies should be monitored for signs of decline that could jeopardise the effectiveness of PCT, systematic monitoring and evaluation is seldom implemented. Drug trials mostly report aggregate efficacies in groups of participants, but heterogeneities in design complicate classical meta-analyses of these data. Individual participant data (IPD) permit more detailed analysis of drug efficacies, offering increased sensitivity to identify atypical responses potentially caused by emerging drug resistance.

**Methodology:**

We performed a systematic literature review to identify studies concluding after 2000 that collected IPD suitable for estimating drug efficacy against STH. We included studies that administered a variety of anthelmintics with follow ups less than 60 days after treatment. We estimated the number of IPD and extracted cohort- and study-level meta-data.

**Principal findings:**

We estimate that there exist individual data on approximately 35,000 participants from 129 studies conducted in 39 countries, including 34 out of 103 countries where PCT is recommended. We find significant heterogeneity in diagnostic methods, times of outcome assessment, and the reported measure of efficacy. We also quantify cohorts comprising pre-school age children, pregnant women, and co-infected participants, including with HIV.

**Conclusions:**

We argue that establishing a global IPD repository would improve the capacity to monitor and evaluate the efficacy of anthelmintic drugs, respond to changes and safeguard the ongoing effectiveness of PCT. Establishing a fair, transparent data governance policy will be key for the engagement of the global STH community.

## Author summary

Soil-transmitted helminthiases (STHs) caused by roundworm, whipworm or hookworm affect over one billion of the world’s poorest people mostly living in low and middle income countries, exerting a major health and economic toll. These infections are controlled by regular mass drug distribution to affected populations. But with very few alternative medicines, the effectiveness of treatment programmes is vulnerable to the potential emergence of drug resistance. Despite a recent scale-up of mass drug distribution, systematic monitoring and evaluation of the efficacy of treatment is too rarely undertaken and our knowledge of how the drugs are performing is largely based on information from clinical trials. However, the design and reporting of information from these trials is very variable which makes it difficult to form a comprehensive picture of the status and trends in drug efficacy. Here, we present a systematic review of published studies completed since 2000, characterise variation in their design, implementation and reporting and estimate the abundance of individual participant data. We argue that the co-ordinated sharing of these individual data would greatly increase the capacity of the global health community to monitor effectively drug efficacy, to respond accordingly to changes, and thereby to safeguard the effectiveness of STH control.

## Introduction

Soil-transmitted helminthiases (STHs) are a group of intestinal nematode infections of humans most commonly, though not exclusively, caused by the roundworm *Ascaris lumbricoides*, the whipworm *Trichuris trichiura* and the hookworm species (*Necator americanus* or *Ancylostoma duodenale*). These infections place a heavy burden of disease on endemic regions, mostly low and middle income countries (LMICs). An estimated 3.2 million years lived with disability (YLDs) were caused by STHs in 2015, with 1.8 million attributed to hookworm infection [1] which is associated with intestinal blood loss, iron deficiency anaemia and protein malnutrition [2]. Ascariasis is the most common STH with a global prevalence estimated at over 760 million. Altogether, it is estimated that about 1.45 billion people—about 20% of the world’s population—suffer from STHs [1].

Soil-transmitted helminthiases are treated and controlled predominantly using the benzimidazole drugs albendazole or mebendazole. The World Health Organization (WHO) recommends giving single (400 mg for albendazole, 500 mg for mebendazole) doses of benzimidazoles to pre-school age children (pre-SAC) and school age children (SAC) in endemic communities (the latter defined as having ≥20% overall prevalence) at least annually as part of the preventive chemotherapy and transmission control (PCT) strategy delivered by mass drug administration (MDA) [3]. This strategy is aimed at controlling and preventing morbidity caused by STHs and, to meet this objective, the WHO has set 2020 treatment-coverage goals for at-risk populations of 75% in pre-SAC and SAC [4]. These goals were endorsed at the 2012 London Declaration on Neglected Tropical Diseases (NTDs) [5] and have since driven a scale-up in the global distribution of benzimidazoles. In 2015 alone, 572.7 million pre-SAC and SAC were treated for STH infection, approximately 60% of the globally at-risk population requiring preventive chemotherapy and a doubling in global treatment coverage since 2010 [6].

The scale-up of MDA towards the 2020 goals is projected to increase greatly the cost-effectiveness of STH control [7]. However, the continuing effectiveness of PCT as a control strategy depends on the enduring efficacy of the distributed drugs. There exist arguments both for and against STH resistance to benzimidazoles emerging [8]. Arguably, use of antimicrobial monotherapy has almost systematically led to emergence of resistance for other infectious disease pathogens, and, with few alternative treatments available or novel anthelmintics in the developmental pipeline [9], the consequences of resistance are potentially severe. In some helminth infections of livestock (particularly sheep), resistance is so widespread and arises so frequently that treatment-based control becomes ineffective [10, 11]. It is essential that responses to treatment are monitored to identify signs of waning efficacy [8, 12–15].

The WHO has issued technical guidance and standardised protocols on monitoring the efficacy of anthelmintic drugs using microscopy-based parasitological diagnostics [16], including a requirement to follow up participants (SAC) 2–3 weeks after treatment to improve homogeneity of data collection. The recommended analytical techniques—largely adapted from methods applied in veterinary medicine where monitoring resistance in herds is commonplace [17, 18]—are based on measuring the average efficacy in groups of children at a population or community level. However, it has been argued that this is an insensitive means of detecting the early warning signs of dwindling drug efficacy and alternative methods based on individual participant data (IPD) [19, 20] have been proposed. Another important limitation of the current system is the added imprecision contributed by the diagnostic methods used (detecting eggs in feces) and the need for more sensitive molecular assays [21, 22]. In practice, few control programmes routinely evaluate drug efficacy because of logistical complications and the additional burden on resources of returning to treated communities before the next round of MDA is due. Knowledge of how the anthelmintics used for MDA are performing is thus largely based on information from clinical trials or other research studies.

The case for collating and sharing IPD has been argued recently in the context of monitoring the efficacy of antischistosomal drugs, particularly praziquantel, which is the mainstay of schistosomiasis control and elimination efforts [3, 4]. A recent landscaping systematic review [23]—and companion to the work presented here—identified more than 20,000 IPD collected globally since 2000. Without these IPD, it is virtually impossible to disentangle the effects of heterogeneous study designs from more meaningful temporal or spatial trends [24]. Hence, only the sharing, standardisation and analysis of IPD will make it possible to evaluate comprehensively global trends in the efficacy of anthelmintics, both against schistosomiasis and STHs.

Here we present a systematic review that identifies studies that have collected IPD on the efficacy of benzimidazoles and other drugs used to treat STH. We evaluate heterogeneity in study designs, geographical location and other study features and we estimate the abundance of IPD, thus evaluating the feasibility and value of establishing a global IPD repository for STH.

## Methods

### Literature search strategy

We carried out a pilot search to estimate the volume of literature and the nature of the studies to be examined in the landscaping exercise. We searched the MEDLINE and Embase databases using a keyword-only search (no MeSH or EMTREE terms) and examined the references for potential inclusion. Based on the results, we decided to exclude search terms pertaining to trials, as there were some studies that were not drug trials but that did collect data from which drug efficacy could be calculated (e.g. in studies testing diagnostic methods).

We developed detailed search strategies for the databases MEDLINE, Embase, Web of Science, and the Cochrane Library and Cochrane Infectious Diseases Group register, compiling a list of disease and parasite-related terms, and a list of drugs. No limits on publication date or language were imposed within the search strategy. Where possible, we used controlled vocabulary terms to filter out non-human based research. In addition, relevant references were identified from bibliographies of other published secondary analyses, including the companion antischistosomal efficacy data landscaping review [23]. The drugs included in the search were: albendazole, mebendazole, levamisole, ivermectin, tribendimidine, nitazoxanide, pyrantel pamoate, and oxantel pamoate. Full details of the search strategy are given in S1 Text. The search was last conducted on 1st July 2016. No contact with authors was attempted at this stage, or during data extraction.

### Screening and inclusion criteria

After automatic and manual de-duplication of the search results, we eliminated those published before 2001 (as a first step to eliminating studies completed before 1^st^ January 2000). This cut-off was applied because older data are generally more difficult to retrieve [25], lowering the likely availability for an of IPD database. Conference abstracts before 2014 were excluded due to increasing probability of secondary reporting on studies published elsewhere. These limits are consistent with the search conducted by Julé et al. [23] on studies generating IPD on antischistosomal efficacy. We screened the titles, keywords and abstracts and/or introductions of the remaining records for inclusion. We excluded: non-human or *in vitro* studies; case reports or series, and retrospective studies; reviews, secondary analyses and other non-primary research; studies in non-endemic settings; studies on costs only, coverage, perception or other aspects of MDA programmes. Results that were not excluded by this screening were retained for full-text reading and are itemized in S1 Dataset.

Full text articles were assessed using a checklist (see S1 Dataset). This checklist confirms whether the data from at least some participants in a study would be suitable for estimating drug efficacy. The checklist includes whether the study: involved diagnosis of STH infection in at least a subset of participants; administered at least one of the drugs in the search (S1 Text) to some infected participants, and carried out post-treatment parasitological diagnosis in some of those participants 60 days or less after the first treatment. This cut-off is longer than the 14- to 21-day timeframe recommended by the WHO [16]. Notwithstanding the potential diluting effect of reinfection on measurements made after 21 days, we adopted a more liberal cut-off to include data that may still be informative on the efficacy of anthelmintics (with suitably adjusted statistical analysis), particularly against hookworm which can have a pre-patent period of 6 to 9 weeks [26, 27].

When we identified multiple reports from one study, all relevant publications were noted, and one was chosen based on the amount of information found in each publication. Other reports were used for confirmation of unclear details or further data extraction if details were not found in the primary reference.

### Standardised data extraction

Two researchers (JBH, JB) extracted data on study: i) setting; ii) design; iii) participant (cohort-level) characteristics; iv) type of outcomes measured and reported in the references; v) the anthelmintic regimens used, and (where reported) vi) the numbers of individual participants treated and followed up. An estimate of the number of IPD per study arm useable for efficacy estimation was made using the reported data (where sufficient) i.e. an estimate of the number of participants diagnosed and tested positive, treated, and followed up within 60 days after treatment. To facilitate standardised data extraction, we adapted a variable dictionary developed by Julé et al. [23], modifying it to fit the requirements of this STH search (S2 Text). Key terms from this dictionary are included in Table 1. We obtained native language support for publications in Chinese, Spanish and Portuguese; data extraction from these publications was conducted in discussion with the researchers extracting the data from the English-language references to ensure consistency.

**Table 1:** Glossary of key terms from the variable dictionary.

Term	Definition
Study	A study or trial, where a pre-defined protocol was followed for all participants, with the exception of differences between cohorts and arms.
Cohort	Groups within studies are considered as separate cohorts if at least one of these reasons applies: there were differences in the protocol followed for different groups of participants (other than those covered by different intervention arms); study recruitment took place in multiple distinct time periods, or participants were in different countries; participants were in *a priori* defined categories which depended on participant characteristics (e.g. diagnosis of different soil-transmitted helminth infections or other co-infections; ethnicity).
Arm	Different arms of a study often correspond to different drug regimens under comparison. Study- and cohort-level data are the same for different arms; only treatments/interventions vary. The arms are such that participants (or clusters, as per design) could be randomized to them (though randomization was not implemented in all studies).

### Estimating the number of individual participant data

We estimated the number of IPD in most instances where it was not clear how many participants’ data could be used to estimate efficacy. This often corresponded to cases where a positive diagnosis was not a criterion for treatment and where not enough data were reported to calculate (rather than estimate) how many of the followed participants were initially positive. The estimation method was adapted depending on the reported items in the publication. Common methods are detailed in S3 Text; full details of the methods used for each study arm are given in S3 dataset. The two most common examples are:

1. If a baseline (pre-treatment) prevalence of STHs caused by the three parasites of interest (*A*. *lumbricoides*, *T*. *trichiura* and hookworm) combined was reported, this was multiplied by the number of participants followed up, making the assumption that loss to follow up was not dependent on parasite-specific infection status.
2. If STH prevalence was reported for each parasite separately, but with no clarification of the prevalence of single or multiple infections, then we used the most common infection prevalence as the most conservative estimate of the number of participants initially positive (if this was only given for the baseline population, then estimation method (1) also applies).

## Results

### Search results, geographical coverage and treatments

The literature search yielded 4,095 results after de-duplication. A total of 2,615 full-text articles published 2001 onwards (i.e., eliminating most studies completed before 1^st^ January 2000), or conference abstracts from 2014 onwards, were selected from these. We screened these 2,615 by reading the titles, abstracts/ introductions, and keywords, rejecting 2,111 clearly-ineligible articles. Articles which were not rejected at this initial screening progressed to a full-text elimination process. The 504 search results evaluated for inclusion at full-text level are detailed in S1 Dataset.

**Fig 1:**
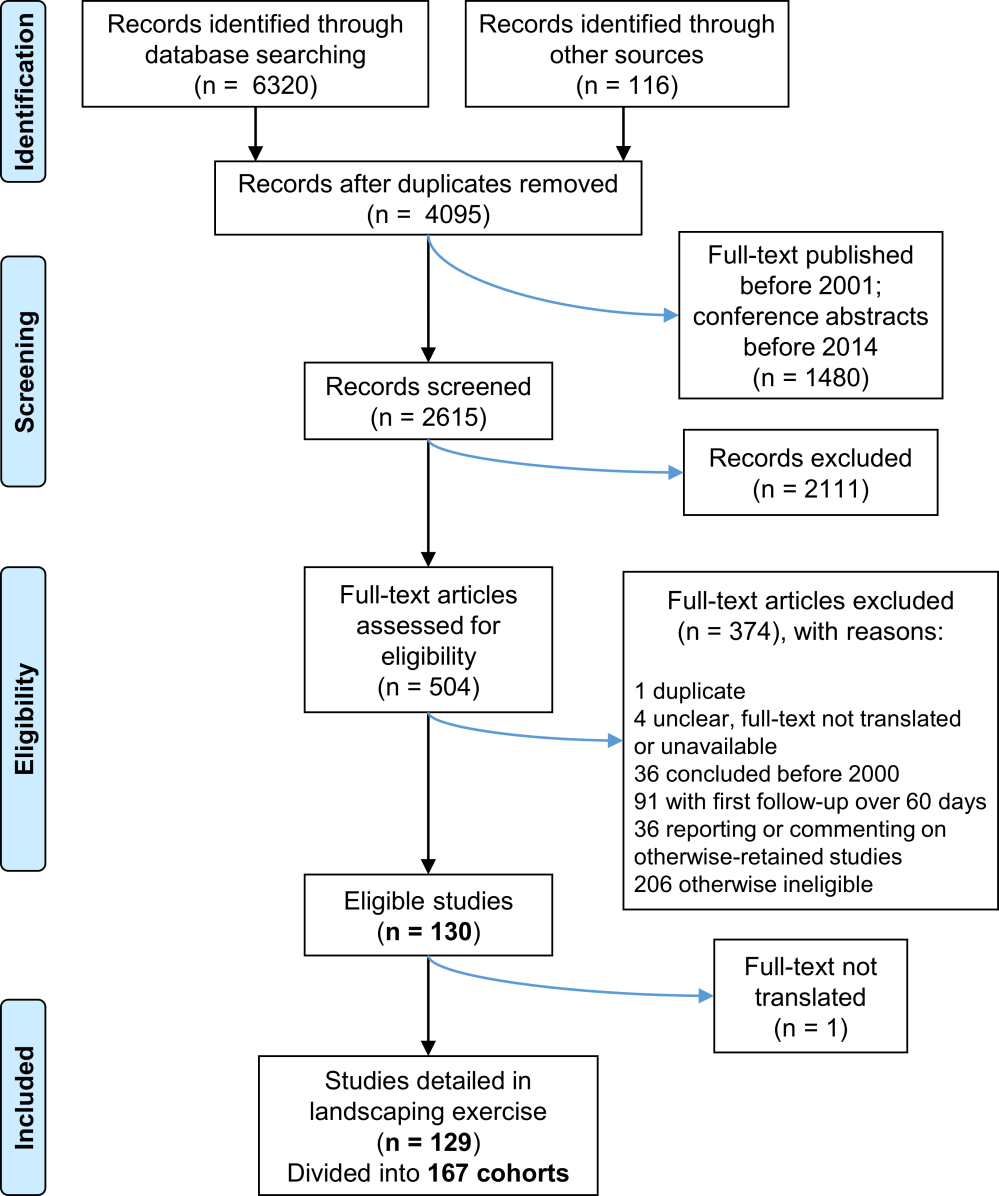
PRISMA flow chart representing the number of items identified in the search and subsequently screened and assessed for eligibility. The search strategy and eligibility criteria are summarised in the Methods section and further details are given in S1 Text.

Having applied the full-text eligibility criteria, we identified 129 studies comprising 167 cohorts for inclusion in this review. Of the 375 excluded search results, 91 were primarily excluded because they had a follow-up visit over 60 days after treatment (but fulfilled other criteria); another 91 did not report assessment of infection after treatment (mainly prevalence surveys, reports of MDA, or on other aspects of treatment such as safety). Five search results had either an inaccessible full text or language support was not available; one of these apparently fulfilled the criteria for inclusion according to the abstract only, but was excluded because the full text could not be checked. The PRISMA flow chart summarising the identification, screening, eligibility and inclusion process is shown in Fig 1.

From the 129 included studies, 167 eligible cohorts were partitioned out and most data extracted at the by-cohort level; data on participant numbers and treatment regimens were extracted per arm. The number of IPD (if given or calculable from the reported items) that could be used for an estimate of efficacy was recorded. In 78 cohorts (comprising 129 study arms), this number was estimated (see methods and S3 Text), and in 15 cohorts, no calculation or estimate was made due to a lack of reported data (usually either variable follow-up time, or no indication of prevalence of STH, especially in studies whose focus was on other parasites). The IPD estimates given within these results therefore represent an estimate using data from 152 of the 167 cohorts, in 114 of the 129 included studies.

**Fig 2:**
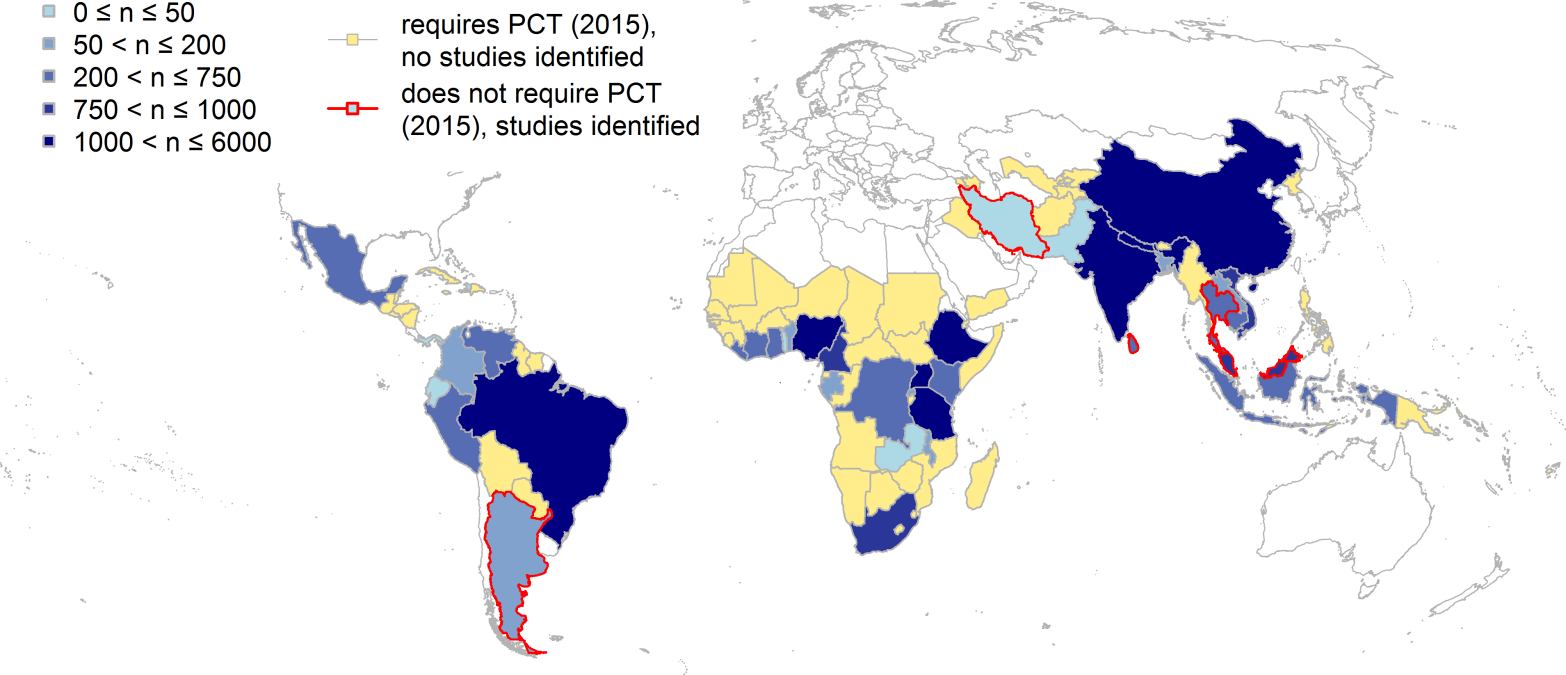
Geographical coverage of studies collecting data on the efficacy of drugs for treating ascariasis, trichuriasis and hookworm. The 39 countries with studies generating individual participant data (IPD) suitable for estimating drug efficacy are shaded blue, with darker shades corresponding to higher estimated abundance of IPD. Five of these, with a red border, do not require preventive chemotherapy and transmission control (PCT) for soil-transmitted helminthiases [24] (infection prevalence <20%). while the other 34 require PCT. The 69 countries shaded in yellow had no studies identified here, and have been designated by the World Health Organization as requiring PCT. The map was created using freely available country outline data from Natural Earth (naturalearthdata.com).

Included studies covered 34 of the 103 countries listed as requiring PCT by the WHO in 2015 [28], with additional studies in 5 countries (Argentina, Iran, Malaysia, Sri Lanka, Thailand) not indicated as requiring PCT (Fig 2). In total, we identified 85 of the 129 included studies as having a drug efficacy assessment as a primary aim of the study. A total of 23 studies included a focus on a parasite other than those causing the three STHs of interest, including 6 with a focus on intestinal schistosomiasis.

The treatment regimens applied in the studies covered a variety of drugs and dosages. Table 2 presents the number of studies and the associated IPD estimates for some common drugs and regimens; full details accompany each study arm’s entry in S3 Dataset. The most common drug administered is albendazole, with mebendazole second; of note, there are also some large trials of chemically-unrelated compounds such as tribendimidine (an aminophenylamidine) [29], several smaller studies administering ivermectin (a macrocyclic lactone) [30], and comparatively smaller trials of nitazoxanide (a thiazolide) [31]. Diethylcarbamazine, not included in the search strategy, was administered (and its effect on STH estimated) in one included study [32] and one excluded study (due to there being no comparator in the list of drugs of interest). Many additional drugs were administered to participants, most notably praziquantel, given to at least 27 cohorts (either as part of the regimens tested or when schistosomiasis was diagnosed).

**Table 2:** Commonly administered drug regimens from the 129 studies collecting data on the efficacy of drugs for treating ascariasis, trichuriasis and hookworm. These regimens account for the majority (89%) of IPD suitable for efficacy estimation. Other miscellaneous drugs or regimens administered include variations on the common mebendazole regimens, levamisole, diethylcarbamazine (DEC), and plant extracts tested for their anthelmintic effect. There are also some treatments which were not reported in enough detail to characterise.

Drug name	Dosage regimen	Number of studies (cohorts) administering this regimen	Estimated number of IPD^1^
Albendazole^2^	400 mg single dose^3^ 400 mg, age > 12 years / 200 mg, age < 12 years, single doses	64 (78) 6 (7)	12,574 1,130
	other “albendazole-only” regimens^4^	25 (25)	3,740
Mebendazole^2^	500 mg single dose^3^	18 (24)	3,868
	200 mg per day for 3 days	9 (11)	948
Tribendimidine	various	6 (9)	2,600
Ivermectin	various, including with other anthelmintics	16 (21)	2,130
Pyrantel / oxantel pamoate, or combination of both	various, including with other anthelmintics	7 (7)	1,838
Nitazoxanide	various, including with other anthelmintics	7 (9)	644
Placebo or untreated or treated with antiprotozoan drugs only		21 (23)	1,477

^1^ individual participant data suitable for efficacy estimation

^2^ alone or with placebo, praziquantel, or antiprotozoan drugs

^3^ World Health Organization recommended dose for mass drug administration

^4^e.g. over 400 mg, and/or multiple doses

### Individual participant data and cohort characteristics

**Fig 3:**
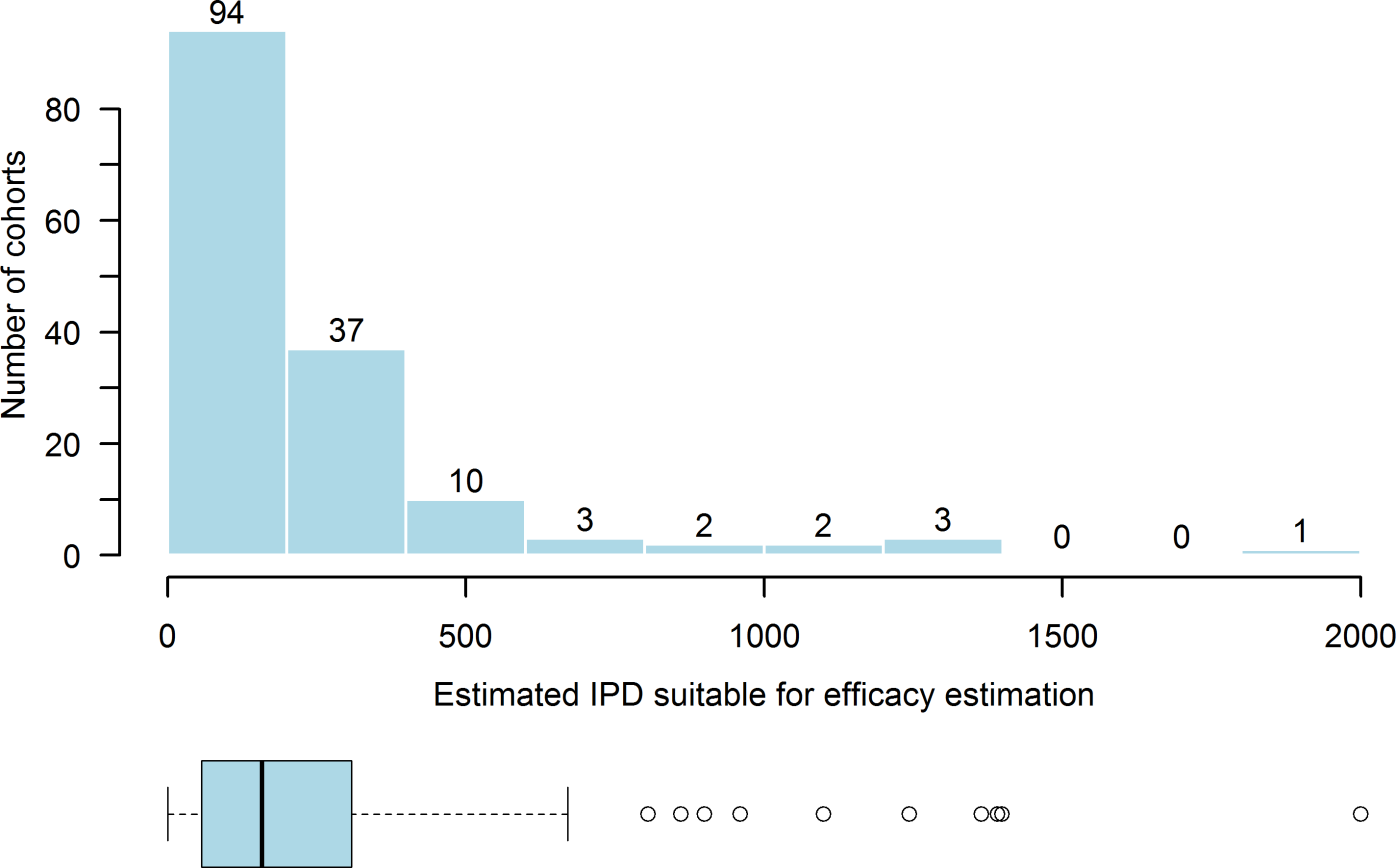
Histogram and box plot showing the right-skewed distribution of the estimated number of individual participant data (IPD) per cohort from 129 studies collecting data on the efficacy of drugs for treating ascariasis, trichuriasis and hookworm. The median estimated cohort size is 146 with a range from 3 to 1,835, with 15 cohorts having no estimate calculated.

We identified 167 cohorts comprising an estimated 35,000 individual participants (diagnosed and testing positive for STHs of interest before treatment and followed up 60 days or less after treatment; see S3 Text for estimation methods) contributing data suitable for estimating drug efficacy for treating STHs. The majority of cohorts comprised fewer than 150 participants, with a heavy right skew to the distribution. 36 cohorts were estimated to comprise fewer than 50 participants eligible for efficacy estimation; WHO guidelines state a minimum of 50 participants should be initially positive (per parasite) if an assessment of efficacy is to be made. (Fig 3). Six cohorts comprised over 1,000 participants. The largest cohorts were found in studies conducted in India [29, 30], comprising 1,835 and 1,283 participants respectively. Generally, most cohorts (92 out of 166, one further study had no information in the conference abstract in which it was reported) focused on SAC, but other demographic groups are represented as shown in Table 3. There were 9 cohorts that included pregnant women only; in the 111 cohorts recruiting any gender and reporting the breakdown, an estimate of 47% of participants were female. The age categories deemed to have been included were judged according to the report of the study. For example, where studies were school-based, the category assigned was SAC, reflecting the intention of the study. In some instances, school age participants were reported to be outside the limits defined by the WHO [6]. In these cases, the minimum and maximum ages reported were extracted but the category was defined as SAC.

**Table 3:** Number of cohorts and estimated number of individual participant data by key age groups from 128 of the 129 studies on the efficacy of drugs for treating ascariasis, trichuriasis and hookworm. Age groups are assigned according to reporting of the study, which does not always coincide with World Health Organization definitions.

Age groups included in cohort	Number of cohorts	Estimated number of IPD^1^
Pre-SAC^2^	6	351
Pre-SAC and SAC^3^	8	2,976
SAC	92	20,772
SAC and adults^4^	21	4,294
Adults, including pregnancy-only cohorts	23	1,680
Pre-SAC, SAC, and adults	16	4,545

^1^ individual participant data

^2^ pre-school age children, as defined or implied in each reference (usually aged ≥ 1 and < 5 years)

^3^ school age children, as defined or implied in each reference (usually aged ≥ 5 and < 18 years)

^4^ as defined or implied in each reference, usually aged > 18 years

### Heterogeneity in study design, methods, and reporting

**Fig 4:**
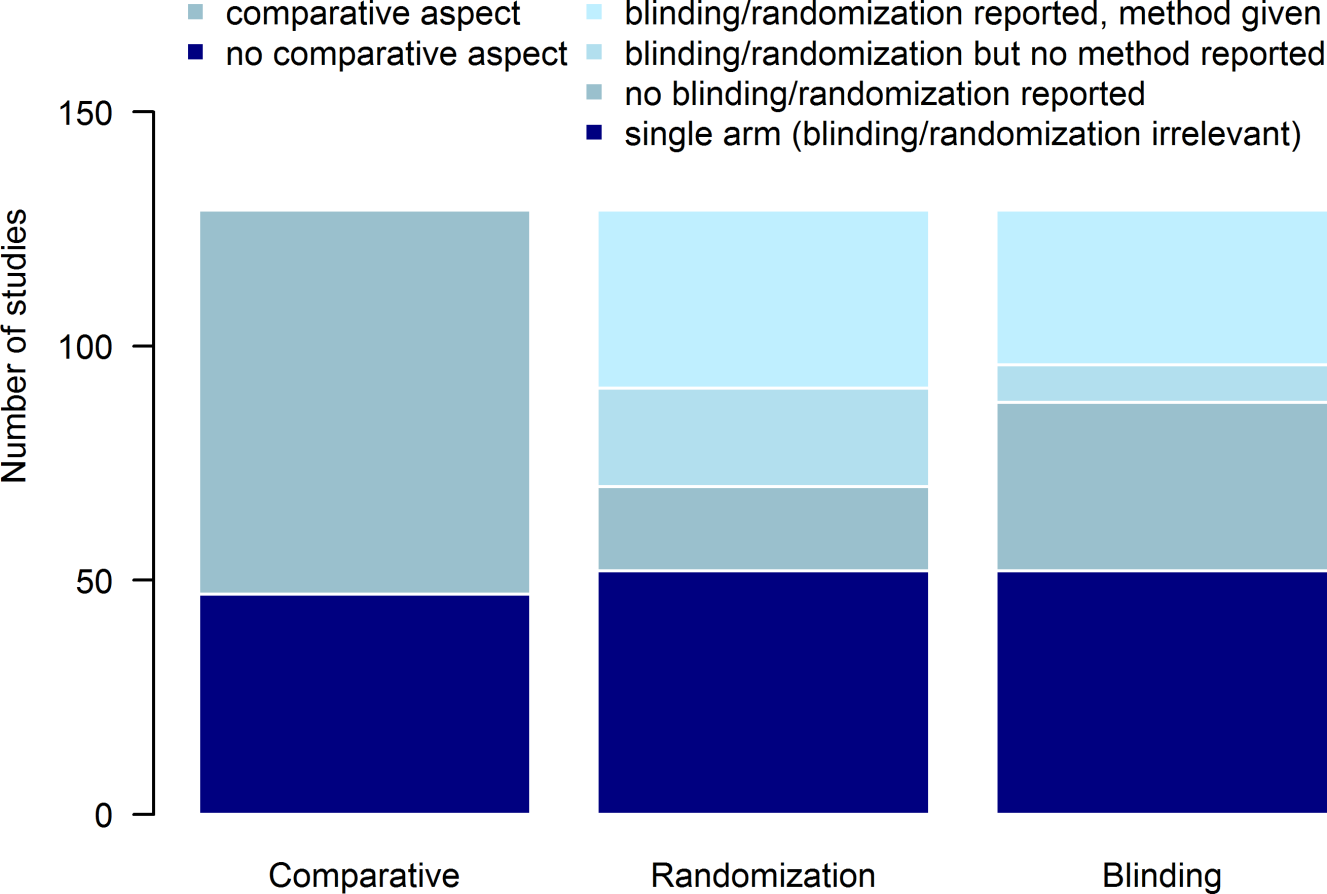
Characteristics of comparative and multi-arm studies collecting data on the efficacy of drugs for treating ascariasis, trichuriasis and hookworm. The left-hand bar shows that of 129 studies, 82 (64%) were assessed as having a comparative aspect. For randomization (middle bar) and blinding (right-hand bar), the bars show (from bottom to top) single arm studies; multi-arm studies in which no randomization/blinding was reported; multi-arm studies where randomization/blinding was reported but with no details of randomization method or of who was blinded, and finally studies where randomization/blinding was reported and details of the method/who was blinded were given.

The majority of studies included a comparative aspect, defined here as either comparing different treatment regimens or comparing treatments in different settings or cohorts (where the comparative aspect was defined *a priori*). For studies comprising more than one arm, not all studies indicated randomization between arms, and for those that did, not all reported the method used. Similarly, blinding was not mentioned (or was not or could not have been carried out) in some multi-arm studies, and for those studies in which it was, the details of who was blinded were not always given (Fig 4). Hence the risk of selection, performance, and detection biases may be difficult to quantify for many studies. Similarly, the number of patients who were lost to follow-up was not always explicitly stated, making risk of attrition bias occasionally troublesome to ascertain.

**Fig 5:**
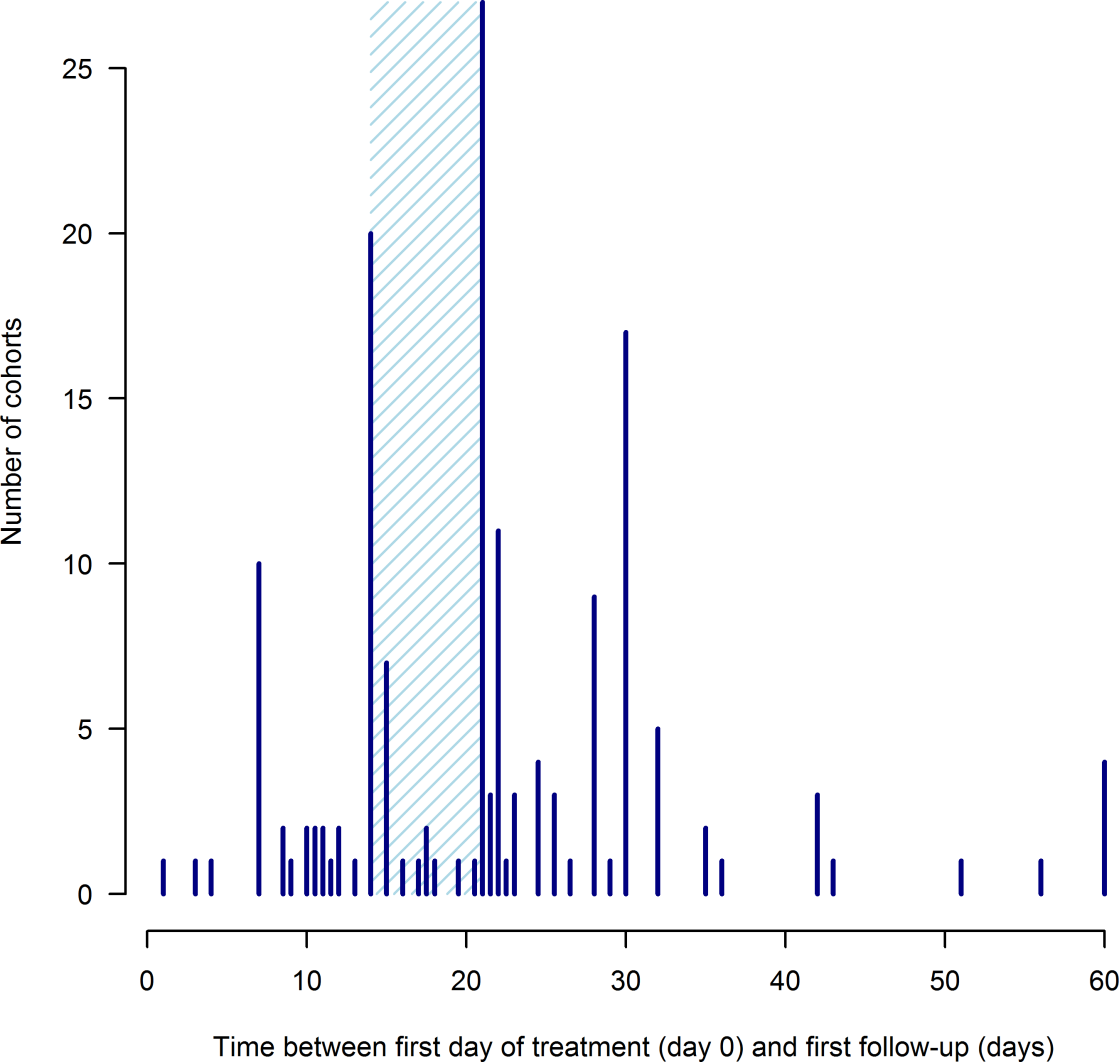
Distribution of cohort follow-up times after treatment from 129 studies collecting data on the efficacy of drugs for treating ascariasis, trichuriasis and hookworm. Only the first follow-up time is included for studies involving multiple follow ups. Treatment is given on day 0. Details on how follow-up time was calculated when a range of values were possible, are found in S2 Text. The shaded area covers 2–3 weeks after treatment, the window over which the majority of studies included here conducted their first follow-up evaluation. Six cohorts had a variable follow-up time and are not included in the figure.

The majority of cohorts were followed up at 2, 3 or 4 weeks after treatment (Fig 5). Two small cohorts had follow-ups under a week only, and 4 cohorts were first followed up at our limit of 60 days.

All studies used microscopy (and occasionally also molecular) methods to detect and count eggs in the feces of individual participants before and after treatment. The Kato-Katz method (or its variations, see S1 Dataset) [33] was the most commonly used diagnostic, albeit a significant number of studies used flotation methods such as the McMaster technique and FLOTAC [34] and a variety of gravity- and solvent-based sedimentation methods [35]. Many studies used multiple methods, sometimes for optimising detection of other parasites or for explicit comparison of diagnostic performance. The techniques used in each study are summarised in Table 4 with further details given in S1 Dataset. Participants in most cohorts were tested for infection using a single replicate from a single stool sample before and after treatment (using the primary diagnostic technique as assigned following the list in Julé et al. [23], with McMaster added to this list). The most tested number of stool samples tested was 3 (before and after treatment) and the most used number of replicates of any one technique per stool sample was 4, albeit the number of sample and replica tests was unclear in a substantial number of cohorts. This heterogeneity is illustrated in Table 5. Participants in 12 of the 167 cohorts were also tested for HIV, and in 20 cohorts for malaria. Reports of 37 cohorts specifically mentioned *Schistosoma mansoni* as a parasite being diagnosed alongside STHs (S1 Fig).

**Table 4:** Summary of main diagnostic methods used to detect and count eggs in the feces of participants in 129 studies collecting data on the efficacy of drugs for treating ascariasis, trichuriasis and hookworm.

Technique	Number of studies (cohorts)	Estimated number of IPD^1^
**Kato-Katz**	** **	** **
41.7, 42, 50 mg	29 (31)	6,490
modified	8 (10)	2,697
unspecified	53 (75)	14,497
qualitative	1 (1)	no estimate for this cohort
**Kato**		
20, 25 mg	2 (2)	401
41.7 mg	2 (2)	1,002
unspecified	3 (3)	259
qualitative	1 (1)	185
**McMaster**		
McMaster or modified McMaster	8 (20)	4,601
**Concentration**		
Concentration (including formaldehyde)	15 (15)	2,135
Other	3 (3)	404
Unclear	4 (4)	1,948

^1^ individual participant data

**Table 5:** Variation in samples and repeats used with the Kato-Katz technique. Number of cohorts (and number of estimated individual participant data in parentheses) from the subset comprising 91 studies collecting data on the efficacy of drugs for treating the soil-transmitted helminthiases ascariasis, trichuriasis and hookworm in which infection was diagnosed, primarily using a Kato-Katz method, before (a) or after (b) treatment by performing *X* × *Y* (samples × replicates) egg counts.

a) Diagnosis		Kato-Katz: replicate slides				
		1	2	3	4	unclear
Samples	1	16 (2,526)	23 (3,969)	5 (1,189)	1 (529)	31 (6,540)
	2	0	14 (3,224)	1 (134)	0	1 (107)
	3	2 (36)	0	0	0	3 (113)
	unclear	0	0	0	0	19 (5,318)
b) Follow-up						
		Kato-Katz: replicate slides				
		1	2	3	4	unclear
Samples	1	15 (3,113)	23 (3,157)	6 (2,775)	1 (529)	27 (6,262)
	2	1 (97)	13 (3,210)	4 (746)	3 (505)	1 (107)
	3	3 (178)	0	0	0	5 (364)
	unclear	0	0	0	0	14 (2,642)

### Efficacy measures

**Fig 6:**
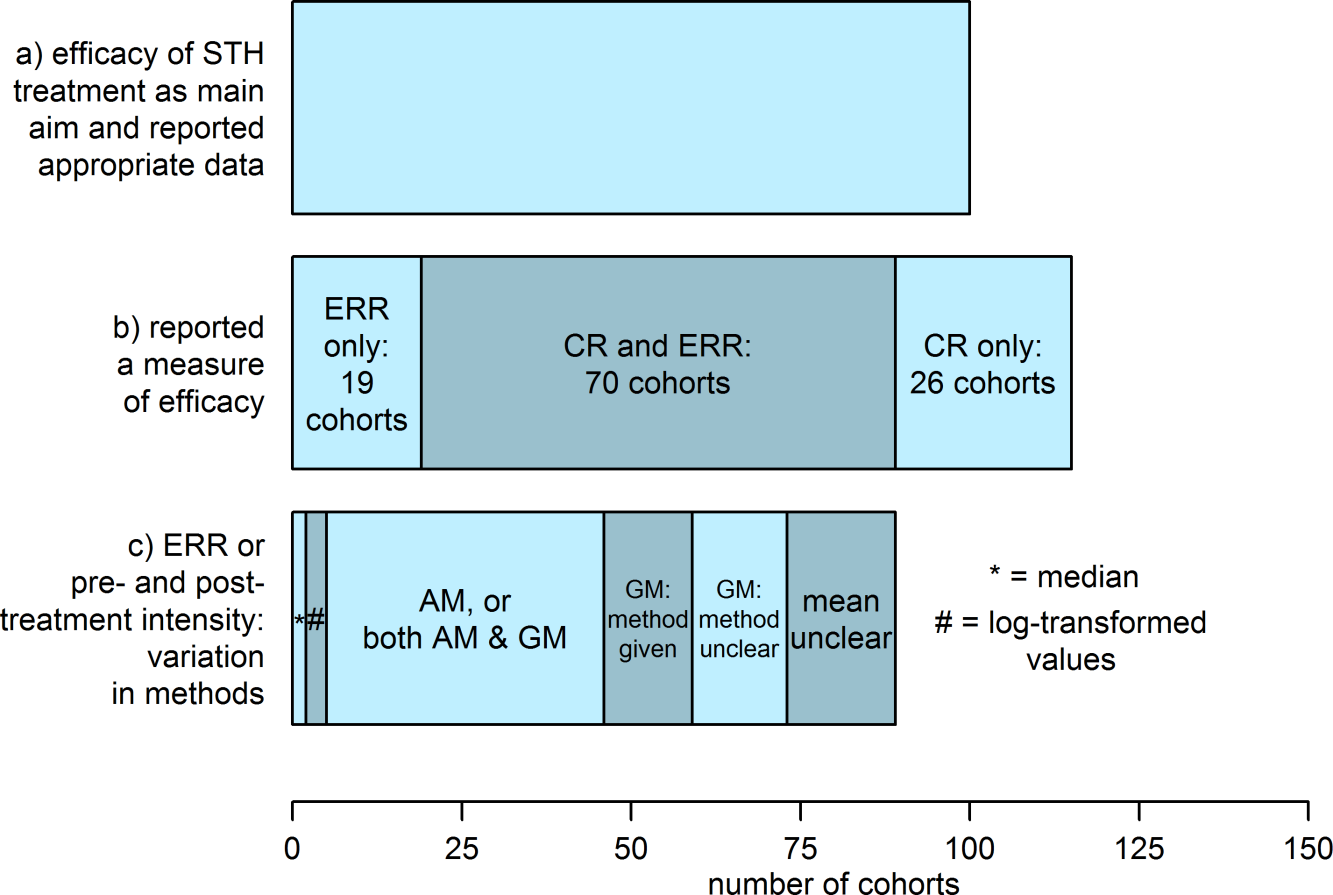
Heterogeneity in efficacy measures reported from cohorts collecting data on the efficacy of drugs for treating ascariasis, trichuriasis and hookworm. Bar (a) shows the total number of cohorts for which measuring drug efficacy against those STHs with a parasitological measure as an outcome was a primary aim. Bar (b) shows the number of cohorts reporting any efficacy measure or appropriate pre- and post-treatment data, divided from left to right into: egg reduction rate (ERR) only; ERR and cure rate (CR), and CR only. Bar (c) shows the total number of cohorts reporting ERR or a pre- and post-treatment measure of intensity, divided from left to right into those: reporting medians; calculating ERR using or reporting log-transformed values; using an arithmetic mean (AM) or both an AM and a geometric mean (GM); using a GM only with a method given; using a GM only with unreported method, and using an unclear measure of central tendency.

For 96 (83%) of the 115 cohorts reporting an efficacy measure, a cure rate (CR), the proportion of participants positive for parasites (or parasite transmission stages) before treatment who become parasitologically negative after treatment, was reported. Many also report an egg reduction rate (ERR), the mean number of eggs per gram of feces (EPG) after treatment expressed as one minus the proportion of the mean EPG before treatment (and the method recommended by the WHO [16]). Of the 89 cohorts for which an ERR was reported or calculable, 41 (46%) used an arithmetic mean (AM) for the EPG (before and after treatment), with the remainder mostly using a geometric mean (GM), albeit with variations on how the latter was calculated. There were 2 cohorts for which a pre- and post-treatment median EPG was reported, and 3 for which either a log-transformed EPG or ERR calculated on log-transformed values was given. Heterogeneity in the reported efficacy measures is illustrated in Fig 6. Within those calculating an ERR-type measure, there is further variety in methods; two studies reported an ERR calculated on uncured participants only (while also reporting a CR). Occasionally an ERR-type measure is calculated using the mean differences between participants’ egg counts pre- and post- treatment, rather than the difference in the group mean.

## Discussion

We present a landscaping systematic literature review of clinical trials and related studies completed since 2000, identifying those that have collected IPD on the efficacy of drugs used to treat the STHs ascariasis, trichuriasis and hookworm. We have estimated that there exist data on approximately 35,000 infected participants who took part in 129 studies across 39 countries trialling a variety of drugs and regimens, including those recommended by the WHO for the control of STHs by PCT. We have uncovered and documented substantial heterogeneities in the design, implementation and reporting of data from these studies. This would make a comprehensive evaluation of the status of anthelmintic efficacy challenging using standard meta-analytical approaches. Yet such evaluations and status updates are becoming increasingly important in the context of the current unprecedented scale-up of MDA for the treatment and control of STHs as endemic countries try to reach the impending 2020 milestones [6]. To this end, for these data to be most useful they would ideally be collated, standardised in a well-designed shared database and pooled for the purpose of conducting IPD meta-analyses.

This study is a companion to a recent similar landscaping review of IPD on antischistosomal drug efficacy [23]. Just as with antischistosomal efficacy trials, heterogeneities in study design (e.g. the use of control cohorts, the randomization of participants or communities to receive different treatments), implementation (e.g. follow-up times for the assessment of efficacy and the demographic groups included in the study) and the methods of measuring and reporting efficacy (e.g. CRs or ERRs using arithmetic or geometric means themselves derived from different calculations) can all influence the efficacy outcome. This makes it extremely difficult to use all the available aggregated information to undertake comprehensive evaluations on the global status of drug efficacy and on understanding potential geographical variation and temporal trends.

Classical meta-analyses of antischistosomal and anti-STH efficacy generally adopt stringent inclusion criteria to ensure data quality and limit the influence of fundamental heterogeneities in study design. Often this means that only data from randomized controlled trials are considered for analysis [36–38], severely restricting the availability of data and failing to circumvent problems associated with other heterogeneities (such as variable follow-up times; diagnostic methods; approaches to efficacy evaluation and recruited demographic groups). More relaxed eligibility criteria combined with contemporary meta-analytical approaches [39, 40] can increase the available evidence base and help to mitigate the influence of study heterogeneities. Nonetheless even these approaches remain fundamentally limited by the aggregate nature of data reporting.

The benefit of IPD is that both individual- and study-level variables can be incorporated directly into statistical analyses while also accounting for unmeasured or unmeasurable random variation among studies, cohorts and individual participants. This permits not only detailed investigation of the influence of individual participant variables on drug responses (e.g. age, sex, time of follow up and co-infection status) but also residual (unexplained) variation among individual drug responses [20, 41, 42]. Such individual level meta-analyses offer greater sensitivity than their aggregate level counterparts to identify suspicious or atypical drug responses [19] that are potentially indicative of, for example, emerging drug resistance, suboptimal dosing in particular sub-populations, medicine quality issues or drug interactions and warrant further follow-up investigation. In particular, variation in parasite drug susceptibility (possibly genetically mediated) cannot be included in such individual patient (host) analyses and thus molecular [43] follow-up analyses of parasitological samples from individuals exhibiting atypical responses would be informative.

Notwithstanding the potential benefits of IPD to identifying and responding to atypical drug responses, more research must be done to differentiate between truly suspicious responses and naturally expected levels of person-to-person variation. Such variation may be driven by both host [19, 42] and parasite factors [44] (including the more refractory nature of some STH species, particularly *T*. *trichiura* and to a lesser extent hookworm species [36]) but may be dominated by error inherent to parasitological (egg count-based) diagnostics [45–47]. Underlying drivers may become somewhat unmasked as more accurate molecular-based diagnostics [21, 22] become more commonplace. It will be important to define distributions of responses based on both parasitological and new molecular diagnostic approaches in populations infected with treatment-naïve parasites [42] to serve as a comparative reference to response distributions in populations exposed to multiple rounds of MDA under the PCT strategy [19]. Equally important, statistical approaches should properly integrate variability in diagnostics (particularly the high variability associated with parasitological diagnostics) into estimates of efficacies with robust associated uncertainty to avoid ‘false alarms’ from spuriously atypical point estimates [20, 42].

Currently there is no incontrovertible evidence of benzimidazole resistance in humans [8, 13, 15]. Some studies have observed poor responses in communities under long-term MDA [48, 49] although no genetic basis for these responses was found [50]. Nevertheless, it would be poor public health practice to ignore the possibility that resistance could emerge, especially since there are few alternatives available now and there remain significant challenges to incentivising commercial investment in anthelmintic drug discovery and development [51, 52]. This means that the effectiveness and cost-effectiveness of PCT [7] is extremely vulnerable to reductions in drug efficacy and there is consensus on the importance of its robust monitoring and evaluation [8, 12–15].

The WHO recommends that assessment of drug efficacy should be conducted in schoolchildren 14 to 21 days after treatment [16]. This presents logistical and resource challenges to programmes that must return to communities shortly after treatment rounds have been distributed and before the next round of MDA is due. Yet the risks posed by reduced efficacy to the sustainability and cost-effectiveness of PCT programs mean that efficacy assessment should be undertaken, at least in sentinel sites. The WHO guidelines [16] on standardizing the assessment of anthelmintic efficacy will hopefully increase the homogeneity of future efficacy studies. We suggest that guidance on conducting analyses using IPD could complement the existing recommended (population-based) protocols and that these should include detailed instruction on robust quantification and reporting of associated estimates of uncertainty.

Maximizing the potential of IPD for global monitoring and evaluation requires broad stakeholder commitment to data sharing, and a framework that protects the rights of patients, data contributors and data users, and ultimately serves the purpose of increasing knowledge and improving health. The WHO has issued three guiding principles for the operation of data sharing during public health emergencies [53], with adapted operating principles suggested for a global-health orientated approach [54]: an explicit ethical and legal framework governing data collection and use; the publication of results from additional analyses in a reasonable timeframe, and the development and publication of terms of data use by platform operators. Examples of data sharing platforms include Flu Informed Decisions (FluID, https://extranet.who.int/fluid/) and the Worldwide Antimalarial Resistance Network (WWARN, www.wwarn.org).

We have focused here on studies generating IPD that could be used to estimate the efficacy of drugs used to treat STHs, following the general approach of the preceding companion paper that identified > 20,000 IPD suitable for quantifying antischistosomal efficacy [23]. The overlapping geographical epidemiology [55] and the closely related methods of quantitative diagnosis (counting eggs in feces, or urine for urogenital schistosomiasis) and methods for efficacy calculation (e.g. ERRs [16]) mean that schistosomiasis and STH would be natural companions in any future shared database. Indeed, many of the studies identified in our search diagnosed and administered treatment for both diseases (S1 Fig, S1 Dataset). Moreover, four of the eligible studies identified by Julé et al. [23] which were also eligible for inclusion (and included) in this study were not identified by the STH-specific literature search because there were no relevant search terms in the title, abstract, keywords, or controlled vocabulary. This illustrates the possibility of retrieving STH-relevant data from studies on epidemiologically-related infections.

Like Julé et al. [23], we included all studies with a follow up within 60 days of drug administration. This includes a wide range of follow ups, many outside of the 2- to 3-week optimum window recommended by the WHO [16] (Fig 5). However, the inclusion of data collected at various follow up times, including less than 1 week when eggs will not yet have been completely cleared from the stool [56, 57] and efficacy will be underestimated, would provide comprehensive information on the dynamics of the drug response, including initial clearance dynamics and longer-term reinfection or repopulation. The effect of follow up time (and other covariates) could be incorporated at the analysis stage offering a means to compare data collected by heterogeneous study designs [42]. Follow up time is a key variable in the interpretation and estimation of drug efficacy and while we wholly concord with WHO’s recommendation to standardize future study designs [16], the reality of past studies is of heterogeneity (in this and other important variables). Rather than discarding data from such studies, we suggest collation of IPD and suitable adjustment for study design at the analysis stage.

The true availability of IPD on drug efficacy against STH is likely to be even greater than the 35,000 participants estimated here. We adopted a conservative approach to the estimation of the abundance of IPD in the 78 cohorts where it was not explicit, and did not calculate an estimate from a further 15, so that we are likely to have underestimated the true value. We did not estimate the abundance of IPD from all studies on pregnant women because it was frequently unclear how many individuals were followed up before the 60-day cut-off; recruitment and treatment tended to be carried out during a wider temporal window (second trimester onwards) and the first follow-up stool sample was often taken at delivery or at an ante-natal visit yielding a variety of follow-up times that often exceeded our 60-day cut-off. A meaningful estimation of pregnant participants whose data could contribute to an efficacy calculation would require an indication of the distribution of follow-up time.

Our estimated abundance of IPD is also likely to represent an underestimate because our search was limited to four databases in which the majority of the literature is published in English. We did not search the regional CNKI (China National Knowledge Infrastructure) or LILACS (Latin American and Caribbean Health Sciences Literature) databases [58], which may contain relevant literature from endemic areas. Our search did yield a number of non-English language results; we noted that two of these studies originally published in Chinese were published 5 years later in English-language journals. Hence, although some studies may ultimately appear in English, there may be a substantial delay between the original publication and the translated version. One study conducted in Uzbekistan [59], was not translated from Russian and its inclusion in the final analysis would have constituted the sole representative of the central Asian region. A further source of STH treatment efficacy data may be found in studies focused on other diseases; the key example is schistosomiasis, but studies treating other helminthiases (especially strongyloidiasis, often treated with ivermectin) or intestinal protozoan infections may also yield suitable data (many studies identified here treated a range of parasitic diseases). Finally, we note that only published literature was searched, most of which was found through the search of online databases; grey literature and other sources of study information could not be retrieved with the methods used.

## Conclusions

Published clinical trials on the efficacy of the drugs used to treat STH are highly variable in their design, implementation, and reporting of results. This heterogeneous landscape, which is common with antischistosomal drug trials, presents substantial challenges to conducting meta-analyses aiming to evaluate, in a comprehensive manner, the performance of anthelmintics drugs in the context of burgeoning MDA programmes in an attempt to meet the WHO 2020 goals of STH treatment coverage globally. Yet, together, these trials and other studies provide an abundance of IPD that, if extracted and appropriately analysed, could minimise the confounding associated with aggregate data and greatly improve the capacity of the global health community to understand naturally-occurring individual variation in responses and distinguish these from atypical or truly suspicious drug responses, potentially indicative of emerging drug resistance. We believe that this capability presents a compelling argument to embrace a data sharing philosophy within the STH, schistosomiasis and wider NTD communities, to develop a shared IPD database and to adopt rigorous individual-level meta-analysis approaches undertaken by conglomerates of stakeholders and for the benefit of public health end-users and health policy decision makers.

## Supporting information

S1 Text Literature search strategy. (DOCX)

S2 Text Variable dictionary. (DOC)

S3 Text Estimating number of individual participant data. (DOCX)

S4 Text PRISMA checklist. (DOC)

S1 Fig Number of cohorts from studies collecting data on the efficacy of drugs for treating ascariasis, trichuriasis and hookworm, including participants with coinfections at time of treatment. Abbreviations from left to right: S. mansoni, *Schistosoma mansoni*; S. stercoralis, *Strongyloides stercoralis*; H. nana, *Hymenolepis nana*; E. vermicularis, *Enterobius vermicularis*; Taenia spp., *Taenia* species; HIV, human immunodeficieny virus.
(TIF)

S1 Dataset Screening results. (XLSX)

S2 Dataset Cohort-level data. (XLSX)

S3 Dataset Arm-level data. (XLSX)

## Abbreviations

AM: arithmetic mean
CNKI: China National Knowledge Infrastructure
CR: cure rate
EPG: eggs per gram of feces
ERR: egg reduction rate
GM: geometric mean
IDDO: Infectious Diseases Data Observatory
IPD: individual participant data
LILACS: Latin American and Caribbean Health Sciences Literature
LMICs: low and middle income countries
MDA: mass drug administration
PCT: preventive chemotherapy and transmission control
PRISMA: Preferred Reporting Items for Systematic Reviews and Meta-Analyses
SAC: school age children
pre-SAC: pre-school age children
STH: soil-transmitted helminthiases
WHO: World Health Organization.
