# Supplementary material for: Systematic review of studies generating individual participant data on the efficacy of drugs for treating soil-transmitted helminthiases and the case for data-sharing
Source: PLoS Negl Trop Dis. 2017 Oct 31;11(10):e0006053. doi: 10.1371/journal.pntd.0006053 (PMC5681297; doi:10.1371/journal.pntd.0006053)
Supplement: S1 Text — (DOCX) [file pntd.0006053.s001.docx]

# S1 Text Literature search strategy

We developed a literature search strategy for each of Embase, Medline, Web of Science, and the Cochrane library and Cochrane Infectious Diseases Group register to identify studies that generate individual-level participant data (IPD) suitable for the estimation of the efficacy of anthelmintic treatment targeting soil-transmitted helminthiases (STHs) caused by roundworm, whipworm, or hookworm. Under the PICO(T) framework, the question was defined as follows.

*Problem* STH in humans caused by roundworm (*Ascaris lumbricoides*), whipworm (*Trichuris trichiura*), or hookworm (*Ancylostoma duodenale* or *Necator americanus*).

*Intervention* Treatment with any, or a combination of: albendazole; mebendazole; levamisole; ivermectin; tribendimidine; nitazoxanide; pyrantel pamoate, or oxantel pamoate (drug treatment does not have to be the main intervention of the study.

*Comparator/control* No comparator or control was necessary for inclusion in this landscape exercise.

*Outcome* Diagnosis of STH before and up to 60 days after treatment.

*Type of study* Any study with a prospectively defined protocol that generated the data of interest; i.e. not limited to clinical trials of the drugs themselves.

The search strategy was constructed using the problem and the intervention only. There are no outcome-related terms which could be included in the search (because the measurement of such outcomes may not be mentioned in the searched fields) and the types of study eligible were diverse. The strategy for each database is detailed below.

| **EMBASE through OVID** | | | |
| --- | --- | --- | --- |
| **Concept and category** | | | **Notes** |
| **Disease terms** | | |  |
| Free-text STH terms | 1 | (soil transmitted adj3 nematod*).mp. |  |
|  | 2 | (soil transmitted adj3 helminth*).mp. |  |
|  | 3 | geohelminth*.mp. |  |
|  | 4 | (intestinal adj3 helminth*).mp. |  |
|  | 5 | (gastrointestinal adj3 helminth*).mp. |  |
|  | 6 | (intestinal adj3 nematod*).mp. |  |
|  | 7 | (gastrointestinal adj3 nematod*).mp. |  |
|  | 8 | 1 OR 2 OR 3 OR 4 OR 5 OR 6 OR 7 | These are free-text terms which may be used to describe infection with soil-transmitted helminthiasis. |
|  |  |  |  |
| EMTREE subject headings – general helminth | 9 | helminthiasis/ | Infection with helminths |
|  | 10 | helminth/ |  |
|  |  |  |  |
| *Ascaris lumbricoides* terms | 11 | ascariasis/ |  |
|  | 12 | Ascaris lumbricoides/ or (Ascaris AND lumbricoides).mp. | Exists as subject heading |
|  | 13 | (roundworm? OR round worm?).mp. |  |
|  |  |  |  |
| *Trichuris trichiura* terms | 14 | trichuriasis/ |  |
|  | 15 | Trichuris trichiura/ or (Trichuris AND trichiura).mp. | Exists as subject heading |
|  | 16 | (whipworm? OR whip worm?).mp. |  |
|  |  |  |  |
| Hookworm terms: 2 species | 17 | hookworm infection/ |  |
|  | 18 | hookworm/ |  |
|  | 19 | ancylostomiasis/ |  |
|  | 20 | necatoriasis/ |  |
|  | 21 | Ancylostoma duodenale/ OR (Ancylostoma AND duodenale).mp. | Exists as subject heading |
|  | 22 | Necator americanus/ OR (Necator AND americanus).mp. | Exists as subject heading |
|  | 23 | (hookworm? or hook worm?).mp. |  |
|  |  |  |  |
|  | 24 | 9 or 10 | general subject headings |
|  | 25 | 11 or 12 or 14 or 15 or 17 or 18 or 19 or 20 or 21 or 22 | subject headings and species names for each |
|  | 26 | 13 or 16 or 23 | common names with plural wildcards |
|  |  |  |  |
|  | 27 | 8 or 24 or 25 or 26 | **All disease terms** |
|  |  |  |  |
| **Drugs** | | |  |
|  | 28 | albendazole/ or albendazole.mp. | Exists as subject heading |
|  | 29 | mebendazole/ or mebendazole.mp. | Exists as subject heading |
|  | 30 | levamisole/ or levamisole.mp. | Exists as subject heading |
|  | 31 | ivermectin/ or ivermectin.mp. | Exists as subject heading |
|  | 32 | tribendimidine.mp. |  |
|  | 33 | nitazoxanide/ or nitazoxanide.mp. | Exists as subject heading |
|  | 34 | oxantel embonate/ or oxantel pamoate.mp. | subject heading uses different term |
|  | 35 | pyrantel embonate/ or pyrantel pamoate.mp. | subject heading uses different term |
|  |  |  |  |
|  | 36 | 28 or 29 or 30 or 31 or 32 or 33 or 34 or 35 | **All drug terms** |
|  |  |  |  |
|  | 37 | 26 AND 35 | **Disease and Drug** |
|  |  |  |  |
| **exclude non-human studies: Embase** | 38 | 36 not ((exp animal/ or nonhuman/) not exp human/) | exclude any indexed to indicate no humans in study |

| **MEDLINE through OVID** | | | |
| --- | --- | --- | --- |
| **Concept and category** | | | **Notes** |
| **Disease terms** | | |  |
| Free-text soil-transmitted helminthiasis terms | 1 | (soil transmitted adj3 nematod*).mp. |  |
|  | 2 | (soil transmitted adj3 helminth*).mp. |  |
|  | 3 | geohelminth*.mp. |  |
|  | 4 | (intestinal adj3 helminth*).mp. |  |
|  | 5 | (gastrointestinal adj3 helminth*).mp. |  |
|  | 6 | (intestinal adj3 nematod*).mp. |  |
|  | 7 | (gastrointestinal adj3 nematod*).mp. |  |
|  | 8 | 1 OR 2 OR 3 OR 4 OR 5 OR 6 OR 7 | These are free-text terms which may be used to describe soil-transmitted helminthiasis. |
|  |  |  |  |
| MeSH – general helminth | 9 | helminthiasis/ | Infection with helminths |
|  |  | (not to use: helminths/) | scope note and indexing guidelines for Medline suggest the similar helminths term will not be used for infection |
|  |  |  |  |
| *Ascaris lumbricoides* terms | 10 | ascariasis/ |  |
|  | 11 | Ascaris lumbricoides/ or (Ascaris AND lumbricoides).mp. | Exists as subject heading |
|  | 12 | (roundworm? or round worm?).mp. |  |
|  |  |  |  |
| *Trichuris trichiura* terms | 13 | trichuriasis/ |  |
|  | 14 | Trichuris/ or (Trichuris AND trichiura).mp. | Trichuris exists as subject heading |
|  | 15 | (whipworm? or whip worm?).mp. |  |
|  |  |  |  |
| Hookworm terms: 2 species | 16 | hookworm infections/ |  |
|  | 17 | ancylostomiasis/ |  |
|  | 18 | necatoriasis/ |  |
|  |  | Ancylostomatoidea/ |  |
|  | 19 | Ancylostoma/ or (Ancylostoma AND duodenale).mp. | Ancylostoma exists as subject heading |
|  | 20 | Necator americanus/ or (Necator AND americanus).mp. | Exists as subject heading |
|  | 21 | (hookworm? or hook worm?).mp. |  |
|  |  |  |  |
|  | 22 | 10 or 11 or 13 or 14 or 16 or 17 or 18 or 19 or 20 |  |
|  | 23 | 12 or 15 or 21 | common names with plural wildcards |
|  |  |  |  |
|  | 24 | 8 or 9 or 22 or 23 | **All disease terms** |
|  |  |  |  |
| **Drugs** | | |  |
|  | 25 | albendazole/ or albendazole.mp. | Exists as subject heading |
|  | 26 | mebendazole/ or mebendazole.mp. | Exists as subject heading |
|  | 27 | levamisole/ or levamisole.mp. | Exists as subject heading |
|  | 28 | ivermectin/ or ivermectin.mp. | Exists as subject heading |
|  | 29 | tribendimidine.mp. |  |
|  | 30 | nitazoxanide.mp. | Exists as subject heading |
|  | 31 | oxantel pamoate.mp. | no subject heading |
|  | 32 | pyrantel pamoate/ or pyrantel pamoate.mp. | Exists as subject heading |
|  |  |  |  |
|  | 33 | 25 or 26 or 27 or 28 or 29 or 30 or 31 or 32 | **All drug terms** |
|  |  |  |  |
|  | 34 | 24 AND 33 | **Disease and drug** |
|  |  |  |  |
| **exclude non-human studies: Medline** | 35 | 34 not (animals/ not humans.sh.) | exclude any indexed to indicate no humans in study |

| **Web of Knowledge: WoS core** | | | |
| --- | --- | --- | --- |
| **Concept and category** | | | **Notes** |
| **Disease terms** | | |  |
| Free-text soil-transmitted helminthiasis terms | 1 | TS = ( ("soil transmitted" near/2 nematod*) OR ("soil transmitted" near/2 helminth*) OR (geohelminth*) OR (*intestinal near/2 helminth*) OR (*intestinal near/2 nematod*) ) | geohelminth is also the French term  Free-text terms which may be used to describe soil-transmitted helminthiasis. |
|  |  |  |  |
| Species names | 2 | TS = ( (Ascaris lumbricoides) OR (Trichuris trichiura) OR (Ancylostoma duodenale) OR (Necator americanus) ) |  |
| Common names | 3 | TS = ( roundworm$ or (round near/0 worm$) or whipworm$ or (whip near/0 worm$) or hookworm$ or (hook near/0 worm$) ) | $ for plural not strictly necessary as WoS searches for plurals anyway |
|  |  |  |  |
|  | 4 | #3 or #2 or #1 | **All disease terms** |
|  |  |  |  |
| **Drugs** | | |  |
|  | 5 | TS = (albendazole or mebendazole) | major, WHO recommended, benzimidazoles |
|  | 6 | TS = (levamisole or ivermectin) | less widely used |
|  | 7 | TS = ("pyrantel pamoate" or "pyrantel embonate" or "oxantel pamoate" OR "oxantel embonate" OR nitazoxanide OR tribendimidine) | much less used drugs / newly tested |
|  | 8 | #7 OR #6 OR #5 | **All drug terms** |
|  |  |  |  |
|  | 9 | #8 AND #4 | **Disease and drug** |
|  |  |  |  |
| **exclude non-human studies: WoS** | 10 | #8 AND #4  Refined by: **[excluding]:** **WEB OF SCIENCE CATEGORIES:** (VETERINARY SCIENCES) | exclude any indexed to indicate no humans in study |

| **Cochrane Library and CIDG register** | | | |
| --- | --- | --- | --- |
| **Concept and category** | | | **Notes** |
| **Disease terms** | | |  |
| Free-text soil-transmitted helminthiasis terms | 1 | (soil transmitted near/2 nematod*) or (soil transmitted near/2 helminth*) or geohelminth* or (*intestinal near/2 helminth*) or (*intestinal near/2 nematod*) | geohelminth is also the French term  Free-text terms which may be used to describe infection with soil-transmitted helminthiases. |
|  |  |  |  |
| MeSH – general helminth | 2 | MeSH descriptor: [Helminthiasis] this term only | Infection with helminths |
|  |  | (not to use: helminths/) | scope note and indexing guidelines for Medline suggest the similar helminths term will not be used for infection |
|  |  |  |  |
| *Ascaris lumbricoides* terms | 3 | MeSH descriptor: [Ascariasis] explode all trees |  |
|  | 4 | MeSH descriptor: [Ascaris lumbricoides] explode all trees |  |
| *Trichuris trichiura* terms | 5 | MeSH descriptor: [Trichuriasis] explode all trees |  |
|  | 6 | MeSH descriptor: [Trichuris] explode all trees | Trichuris exists as subject heading |
| Hookworm terms: 2 species | 7 | MeSH descriptor: [Hookworm Infections] explode all trees | covers [ancylostomiasis] [necatoriasis] |
|  | 8 | MeSH descriptor: [Ancylostomatoidea] explode all trees | covers [Ancylostoma] and [Necator americanus] |
|  | 9 | #2 or #3 or #4 or #5 or #6 or #7 or #8 |  |
|  | 10 | (Ascaris lumbricoides) OR (Trichuris trichiura) OR (Ancylostoma duodenale) OR (Necator americanus) |  |
|  | 11 | roundworm* or (round next worm*) or whipworm* or (whip next worm*) or hookworm* or (hook next worm*) | common names with plural wildcards |
|  |  |  |  |
|  | 12 | #1 or #9 or #10 or #11 | **All disease terms** |
|  |  |  |  |
| **Drugs** | | |  |
|  | 13 | MeSH descriptor: [Albendazole] explode all trees | Exists as subject heading |
|  | 14 | MeSH descriptor: [Mebendazole] explode all trees | Exists as subject heading |
|  | 15 | MeSH descriptor: [Levamisole] explode all trees | Exists as subject heading |
|  | 16 | MeSH descriptor: [Ivermectin] explode all trees | Exists as subject heading |
|  | 17 | MeSH descriptor: [Pyrantel Pamoate] explode all trees |  |
|  | 18 | albendazole or mebendazole | major, WHO recommended, benzimidazoles |
|  | 19 | levamisole or ivermectin | less widely used |
|  | 20 | tribendimidine or nitazoxanide or (pyrantel pamoate) or (oxantel pamoate) or (pyrantel embonate) or (oxantel embonate) | much less used drugs / newly tested |
|  |  |  |  |
|  | 21 | 25 or 26 or 27 or 28 or 29 or 30 or 31 or 32 | **All drug terms** |
|  |  |  |  |
|  | 22 | 24 AND 33 | **Disease and drug** |
