# Supplementary material for: Systematic review of studies generating individual participant data on the efficacy of drugs for treating soil-transmitted helminthiases and the case for data-sharing
Source: PLoS Negl Trop Dis. 2017 Oct 31;11(10):e0006053. doi: 10.1371/journal.pntd.0006053 (PMC5681297; doi:10.1371/journal.pntd.0006053)
Supplement: S2 Text — (DOC) [file pntd.0006053.s002.doc]

S2 Text Variable dictionary

The information extracted from the identified publications is described and coded by the variables listed here, corresponding to the three datasets S1, S2, and S3, and falling into the following categories:

**S1 Dataset Elimination data**

0. Elimination dataset: search results after screening

0.1 Publication reference and source

0.2 Full-text elimination process

**S2 Dataset By-cohort data**

1. Design

1.1 Study identification

1.2 Study objectives

1.3 Summary of comparative aspects and arms in study

1.4 Bias risk: randomisation and blinding

2. Setting and reporting

2.1 Where was the study conducted/cohort recruited?

2.2 When was the study conducted and reported?

3. Outcomes

3.1 What (standard) analyses were performed to diagnose intestinal helminths?

3.2 What other tests were performed to diagnose and characterise soil-transmitted helminthiasis (STH), as well as other biomedical assays, thus providing additional outcome measures

3.3 When were participants followed-up for outcome assessment(s)?

3.4 How was the primary, drug efficacy endpoint expressed?

4. Participant characteristics

4.1 STH parasites of interest: inclusion/exclusion criteria

4.2 What major co-infections were controlled for, and were there related inclusion/exclusion criteria?

4.3 Age range and pregnancy testing/inclusion/exclusion of enrolled participants

4.4 Specific inclusion criteria

4.5 Specific exclusion criteria

**S3 Dataset By-arm data**

5. Numbers of participants

6. Treatment regimens administered

# Elimination dataset: search results after screening

The subset of references from those identified from the literature search **which remain after summary-level screening**. Checklist and explanations for inclusion/exclusion from subsequent analyses.

## 0.1 Publication reference and source

| **X_ID** | Unique identifier given to the reference at full-text reading. This identifier X enables linkage between datasets.  NB: A reference may require several rows to capture information on all cohorts and arms. A cohort identifier is assigned to each cohort at the by-cohort data extraction stage. The X_ID and X_cohort are concatenated in analysis to form unique identifiers for each cohort. A further “arm” identifier (X_arm) is added at the per-am data extra extraction stage. |
| --- | --- |
| **X0_authors, X0_pubYear, X0_pubTitle, X0_pubJournal, X0_pubDOI** | Reference of the journal article or conference abstract, including all authors and full title, as exported from reference manager (Endnote X7). |
| **X0_refType** | Type of reference: “**conference abstract**”, “**journal article**”, with extra text indicating if the full text of a journal article was not accessible or was not translated. |
| **X0_lang** | Language in which full text is written |
| **X1_source** | Factor variable, indicating the source(s) of the reference (separate multiple sources with) “**;**”   - “**search**” – the literature search conducted in this study; - “**published secondary analysis**” – reference found in the bibliography of the published secondary analysis, Keiser and Utzinger 2008[[1]](#footnote-2) used as a source and manually screened for references; - “**Schisto**” – the papers eligible for inclusion in Julé et al 2016[[2]](#footnote-3) (these would already have been checked for a follow up within the required timeframe). |

## 0.2 Full-text elimination process

| **X2_elimReason** | Factor variable, briefly describing primary category into which reference falls for excluding the reference (or “**none**” if reference is retained). Multiple reasons (if >1 is considered equally important) separated by “**;**”. Further information on exclusion given in **#notes** column for this section. |
| --- | --- |
| **X3_treat** | Dummy variable (**1** = Yes/**0** = No). The study involved the delivery of an anthelmintic drug (from the list given in S1) to human participants. Of note, the study may have taken advantage of episode(s) of mass drug administration for drug delivery to participants. The drug delivery may have been as a control group or a pre-treatment for a different intervention. **On their own**, interventions other than drugs aiming to treat infection with *Ascaris lumbricoides*¸ *Trichuris trichiura*, or hookworm (e.g. shoe-wearing, clinical management, especially to mitigate symptoms) are out of scope. |
| **X4_pre (respectively, X4_post)** | Dummy variable (**1** = Yes/**0** = No). The study involved screening of participants for infection with one of the 3 soil-transmitted helminthiasis parasites of interest (*Ascaris lumbricoides*, *Trichuris trichiura*, or hookworm) shortly before (resp., after) delivery of the intervention (baseline survey and individual follow-up, respectively). The screening procedure should involve a recognised diagnostic test (usually egg count in stool).  Isolated prevalence surveys, as well as studies for which the pre- and post- treatment surveys are likely to comprise different participants in a way such that individual participant data (IPD) availability would not enable identification of eligible IPD, are out of scope (“**0**”). |
| **X5_inTime** | Dummy variable (**1** = Yes/**0** = No). At least one outcome assessment (post-treatment diagnostic test) occurred within 60 days (including, exactly 60 days or 2 months) after treatment, where administration of the first study drug counts as Day 0. |
| **X6_eligible** | Auto-filled variable, establishing whether the study is overall eligible. Equals **1** (eligible) if and only if **X3_treat** + **X4_preTest** + **X4_postTest** + **X5_inTime** = 4 and **X2_screen** = “none”; otherwise, equals **0**. |
| **X7_others** | Text variable, listing references that are, as far as can be ascertained, other reports of the same study which are in this full-text elimination set, or the names of large studies of which this report forms a part. List X_ID(s) if the other report(s) is included in this set; if part of those part of a large named study have the acronym or identifier (e.g. “EMaBS” or “Yen Bai”) for the study reported. Reasons for excluding/including potential duplicates are discussed in **#notes** (see below). |
| **X5_timingNotes** | factor variable, listing any reasons why the time of follow-up is not clear or otherwise noteworthy. List all relevant of: “**varying follow-up**”; “**pregnancy**”; “**probably in time (checked for eradication of infection)**”; “**very short**” |
| **X8_status** | Clarification on any which had issues with elimination decision. |
| **X5_timeFirstFU** | Time of first follow-up of eligible studies or of studies which would be eligible if the follow-up was under 60 days. This field is a check to aid X5_inTime decision. |

| **#notes** | Free text, 1 new line per dataset requiring comment(s).  Comments on problems encountered during analysis and coding of the article (missing or unclear data, impossibility to record the data in the desired format, inconsistency between different sources, etc.). Comments may also include details on reasons for inclusion/exclusion of studies whose eligibility was difficult to assess. Leave blank if no comment is required. |
| --- | --- |

# Design

## 1.1 Study identification

| **X0_authors, X0_pubYear, X0_pubTitle, X0_pubJournal, X0_pubDOI,**  **X_ID** | Publication details and identifier assigned in elimination dataset. |
| --- | --- |
| **X_cohort** | Unique cohort identifier given as soon as a new cohort (set of participants) is added to the dataset. See **#cohortsJustif** for details on breaking down studies into cohorts. |
| **A1_trialID** | Trial identifier, if the trial was registered and registration was mentioned in the publication, in ClinicalTrials.gov, Controlled-Trials.com and World Health Organization International Clinical Trials Registry Platform (ICTRP) registries. Enter “**not found**” otherwise.  In alphabetical order of registry, separated by semi-colon if several registries apply. |
| **A1_trial Registry** | Factor variable, corresponding to the name of the trial registry: “**ClinicalTrials.gov**”; “**Controlled-Trials.com (BioMed Central)**”; “**Pan African Clinical Trials Registry (PACTR)**”. Enter “not found” otherwise.  In alphabetical order and separated by semi-colon if several registries apply. |
| **#studyTitle** | Scientific title of the study/trial, as stated at registration. |
| **#cohortsJustif** | Free text: justification for counting a single ‘study’ reported in this article/abstract as several ‘cohorts’ = set of participants sharing the same study meta-data characteristics.  Multi-country trials are divided into a cohort for each country. Trials split temporally, with different participants reported for each time period (e.g. multi-year trials) are divided into a cohort for each time period. Any differences in protocol, not covered by the study arms, indicate division into cohorts. Finally, any groups which are defined, *a priori*, in a way that participants/clusters could not be randomised to them, for example by participant ethnicity or parasites detected at baseline, are considered separate cohorts.    **NB: ages.** Where a drug is dosed in an age-related manner, and ages are not treated *a priori* as separate groups, different age groups having different drug doses are not treated as separate cohorts. The relevant treatment arm is considered to include all ages, since the intention is not to separate a cohort of different ages for comparison. This reflects study designs where participants are assigned to arms and the drugs are administered with the intention of comparing the arms, rather than comparing the subgroups of different ages. In effect, by-age dosing is taken in the same fashion as by-weight dosing. |

## 1.2 Study objectives

| **A2_efficacy** | Dummy variable (**1** = Yes/**0** = No). Was the study designed primarily to assess efficacy or effectiveness of treatment, on any of the infections in the study, by any outcome measure?  Some studies may have been conducted for another purpose (e.g. diagnostic sensitivity assessment, immunology studies), in which case they score “**0**”; but they still collected data that would enable drug efficacy assessment in secondary analyses. |
| --- | --- |
| **A2_safety** | Dummy variable (**1** = Yes/**0** = No). Did the study assess the safety or tolerability of the treatment (i.e. collected data on side effects)? |
| **A2_pharma** | Dummy variable (**1** = Yes/**0** = No). Did the study involve some pharmacokinetics/pharmacodynamics (PK/PD) measures? |
| **A2_allergy** | Dummy variable (**1** = Yes/**0** = No). Were allergies/asthma a focus of the study? |
| **A2_non-drug** | Do the primary aim(s) of the study include investigating a non-drug intervention e.g. Water Sanitation and Hygiene (WASH), Health Education and Learning Package (HELP), shoe-wearing? Free text including the type of non-drug intervention. Plant extracts are considered “non-drug” if they are not produced and delivered in a way that the dosage of the active ingredient can be quantified. |
| **A2_otherParasite** | Do the primary aim(s) of the study include investigation of a parasite other than the three of interest e.g. *Schistosoma* spp*., Strongyloides stercoralis*, *Taenia* spp. Free text including the parasite name. |
| **A2_HIV** | Dummy variable (**1** = Yes/**0** = No). Was the aim primarily to investigate HIV+ patients? |
| **A2_other** | Factor variable, describing other objectives of the study, namely:   - “**diagnostic approach**”: diagnostic test study (e.g. sensitivity assessment), including search for biomarkers of infection which could potentially lead to new approaches to diagnostics; - “**immunological responses**”: assessment of specific antibody responses, etc. - “**nutrition**”: impact of nutrient supplementation on treatment efficacy or other outcomes (e.g. performance at school).   If several levels apply, enter them in alphabetical order, separated by “;”. Enter “none” otherwise. |
| **#objComment** | Free text: further information to clarify study objectives if necessary. |

## 1.3 Summary of comparative aspects and arms in study

| **A3_comparative** | Dummy variable (**1** = Yes/**0** = No). Was the **drug intervention** assessed in a comparative manner?  If study has > 1 arm, this scores “**1**”.  Some studies are formally considered ‘non-controlled’ (arms = 1) but they include a ‘comparison’ of the efficacy of the drug treatment (e.g. between age groups/settings), in which case they also score “**1**” for our analysis. |
| --- | --- |
| **A3_ctrlCat1, 2** | Factor variable, describing the category of comparator used in controlled studies.   - “**drug comparison**”; “**dose comparison**”; “**regimen comparison**” – comparison of anthelmintics and regimens against each other; - “**placebo**”; “**delayed treatment**”; “**untreated controls**”; “**healthy controls;**” - “**other** (**endemic context**, **season**, **treatment history**, or **education**)” – e.g. “other (endemic context)”, for a comparison between low/high or new/old focus of transmission.   If several levels apply, record them in the order suggested above. Enter “unclear” otherwise, or “NA” for non-controlled studies and in superfluous columns.  NB: studies falling only into the category “other” are rather ‘comparative’ than ‘controlled’ studies (see **A3_ comparative**). |
| **A3_studyArms** | Total number of arms in the study. An arm, as opposed to a cohort, is a comparative group within the study which participants/clusters could be randomised to – i.e. inclusion in that arm vs. others is not decided by some intrinsic property of the participant such as age. Usually corresponds to different drugs or regimens being compared. |
| **A3_drugs** | anthelmintic drugs in each arm of the study, in this list: albendazole (**alb**), mebendazole (**meb**), levamisole (**lev**), ivermectin (**ivm**), **tribendimidine**, **nitazoxanide**, **oxantel** **pamoate**, **pyrantel** **pamoate**. Separate arms by “**;**”. If a combination is administered in an arm, use “**+**”. Example entry: alb; alb+ivm; alb+pryantel pamoate. |
| **A3_addpzq** | Dummy variable (1 = Yes/0 = No). Was praziquantel administered in combination with an anti-STH drug as a treatment arm or to any participant receiving an anti-STH drug but who was also infected with SCH? |
| **#armComment** | Comment on unusual designs. |

## 1.4 Bias risk: randomisation and blinding

| **A4_random** | Dummy variable (**1** = Yes/**0** = No). For multi-arm studies, was the assignment to one arm or the other randomised? If not explicitly stated, the assumption is that the study was not randomised. If randomisation is stated with no method reported, this is 1, but the next column A4_randMethod is “unclear”. |
| --- | --- |
| **A4_rand Method** | Semi-free text: method of randomisation into the study if applicable.  (e.g. “**computer-generated sequence**”). Enter “unclear” otherwise, or “**NA**” for non-randomised studies and for studies where randomisation is not applicable (i.e. single-arm studies). |
| **A5_blind** | Dummy variable (**1** = Yes/**0** = No). For multi-arm studies, was the person delivering the drug and/or assessing the participant blinded to the intervention administered? If not explicitly specified or if drugs were distinguishable, the assumption is that the study was not blind. |
| **A5_blind**  **Level** | Factor variable, describing who is blinded in the study if applicable: “**participants**”, “**investigators**”, “**clinician**”, “**drug administrator**”, “**field staff**”, “**staff**”, “**stool analysis**”, “**outcome assessors**”, “**statistician**”.  Enter “unclear” otherwise, or “NA” for non-blinded studies (this includes those with one arm). Add descriptors as necessary to facilitate data extraction. |
| **#biasComment** | free text indicating any complications / issues in blinding and/or randomisation |

# Setting and reporting

## 2.1 Where was the study conducted/cohort recruited?

| **B0_country** | Full name of the country of study. Use UN classification (available at <http://unstats.un.org/unsd/methods/m49/m49regin.htm>). |
| --- | --- |
| **#siteLocation** | Free text: most precise name and address of each and every site of recruitment, including longitude and latitude GPS coordinates, if available. |
| **B1_siteCat** | Factor variable, describing the category of the site chosen as a recruitment centre of target participants: “**school**”; “**hospital/health-centre**”; “**village**”; “**other**” (village/city, households, etc.). Enter “unclear” otherwise. |
| **B1_setting** | Factor variable, describing the setting where recruited participants live: “**rural**”; “**semi-urban**”; “**urban**”. Only enter if this can be ascertained in text. If several apply, enter them in increasing level of urbanisation, separated by “+”.  Enter “unclear” otherwise. |
| **B0_region** | World region of the country of study. Use UN classification (available at <http://unstats.un.org/unsd/methods/m49/m49regin.htm>). |
| **B0_income** | Income group of the country according to World Bank (available at <http://data.worldbank.org/country>). |
| **B2_species** | Factor variable, describing the soil-transmitted helminthiasis (STH) parasites of interest at the study site.  Enter, separated by semicolons, using abbreviations of species, using HW if species of hookworm is not mentioned; i.e. enter those applicable of **Al**; **Tt**; **Ad**; **Na**; **HW.** |
| **B2_speciesCom** | Free text: comments relating to STH species e.g. level of infection, species of hookworm. |

## 2.2 When was the study conducted and reported?

| **B3_studyStart_YYYY /MM, studyEnd_YYYY/MM** | year/month of start/end of study, as appropriate. |
| --- | --- |
| **B5_reportDate_YYYY / MM** | Year/month, respectively of publication (**earliest known**, i.e. chose ‘ePub ahead of print’ date if available), or of presentation at a conference. |
| **B5_firstReportDate_YYYY / MM** | Year/month that study was first reported (if main reference used is a later paper). |
| **B5_addReporting** | X_ID for any additional reporting of study identified in search results and not rejected at screening. |
| **B5_reportMeans** | Factor variable, describing the medium with which research results were reported: “**journal article**”; “**conference**”.  NB: Only conference abstracts from 2014 onwards were analysed; it is assumed that studies presented at conference beforehand have been published by now and are therefore captured elsewhere. |

# Outcomes

## 3.1 What (standard) analyses were performed to diagnose intestinal helminths?

| **#intestinal Standard** | Semi-free text: description of the sample collection strategy and of the laboratory test performed to diagnose infection with STH – e.g. “aim for 3 samples but 2 tolerated; 2 repeats; Kato-Katz thick smears (cellophane, 50 mg)”. Enter “none” if no diagnosis of intestinal helminths by egg count was performed. |
| --- | --- |
| **C0_iSamp_base** | Minimum number of independent stool samples collected (and required for inclusion in the study) to perform diagnosis at baseline. Enter NA if unclear; enter 0 if no test was performed. |
| **C1_iRep_base** | Minimum number of laboratory test repeats made on one same sample to perform diagnosis at baseline. Enter NA if unclear; enter 0 if no test was performed. |
| **C0_iSamp_fu** | Minimum number of independent stool samples collected (and required for inclusion in the study) to perform diagnosis at follow-up(s) up to 60 days. Enter NA if unclear; enter 0 if no test was performed. |
| **C1_iRep_fu** | Minimum number of laboratory test repeats made on one same sample to perform diagnosis at follow-up(s) up to 60 days. Enter NA if unclear; enter 0 if no test was performed. |
| **C3_iTech1, 2** | Factor variable, describing the technique employed to analyse stools.  “**Kato-Katz thick smears (XX mg)**”; “**Kato-Katz method** (= technique, test)”; “**Kato-Katz smears (modified)**”; “**Katz thick smears**”; “**Kato thick smears (XX mg)**”; “**Kato technique (qualitative)**”; “McMaster”; “McMaster (modified)”; “**concentration (formaldehyde)**”; “**hatching test**”; “other”. Information in brackets may or not be recorded depending on it being detailed in the full text or not. If >1 apply, record in the order suggested above, putting the “main” method into **C3_iTech1** and others into **C3_iTech2**, separated by semicolons. Enter “NA” in second column if only one test was performed. |

## 3.2 What other tests were performed to diagnose and characterise soil-transmitted helminthiasis, as well as other biomedical assays (thus providing additional, secondary outcome measures)?

| **C4_testClinic** | Semi-free text: Additional point-of-care (POC)/rapid test or clinical assessment to diagnose and characterise STH (including symptoms); mainly biopsy, dipsticks/reagent strips for haematuria, proteinuria or leukocyturia, visual aspect of excreta, POC circulating antigen, ultrasonography. Enter “none” otherwise. |
| --- | --- |
| **C4_testLab** | Semi-free text: Additional molecular biology assay performed to diagnose and characterise STH infection, mainly ELISA and PCR. Precise the source of biological material (blood, stool, urine…), and the type of molecules assessed in molecular assays (e.g. helminth DNA, soluble egg antigen…). Enter “none” otherwise. |
| **C5_other Assays** | Factor variable, describing other biomedical assays: “**Hb concentration**” (haemoglobin concentration); “**other serology/immunology assays**” (serology for HIV/AIDS diagnosis, cytokines’ profile after peripheral blood monocyte (PBMC) culture, etc.); “**other blood analyses**” (e.g. haematocrit, iron absorption).  Enter “none” otherwise. |

## 3.3 When were participants followed-up for outcome assessment(s)?

| **D1_follow**  **UpTime** | Time of follow-up(s), in days, calculated from the day of first treatment dose (Day 0), up to the day of first re-sampling (decimal fractions allowed).  If the time is specified in weeks, months, or years in the publication, use 1 week = 7 days, 1 month = 30 days and 1 year = 365 days to convert. Enter all follow-up times in protocol, separated by “**;** “.   - If follow-up time states e.g. “12-14 days”, enter mean of the days given. - If treatment is given over a number of days, unless clearly specified which day the follow-up is relative to, add the follow-up time to the number of treatment days. e.g. for a 5 day treatment and a follow-up of one week after treatment the follow-up time estimated here is 11 days. - If different treatment regimens in study last for different lengths of time, add the follow-up time to each and calculate the mean follow up time e.g. for a single treatment, 3 and 5 day treatments and a follow-up time of 14 days after treatment, the mean is 16 days. |
| --- | --- |
| **#followUp_Comment** | Free text: additional notes explaining follow-up times. |

## 3.4 How was the primary, drug efficacy endpoint expressed?

| **D2_effReport** | Factor variable. What efficacy measures were **reported** **suitable for efficacy estimation of anthelmintic activity against STHs of interest**? I.e. which, if any, of cure rate (CR), egg reduction rate (ERR), or pre- and post- treatment intensity (intens), were reported, based on data from a follow-up up to 60 days post-treatment. Enter all relevant of “**CR**”, “**ERR**”, “**intens**” separating multiple entries by “**;** “ or “**none**”. |
| --- | --- |
| **D3_CR** | Dummy variable (**1** = Yes/**0** = No). Was the cure rate (CR) calculated at any time of follow-up, or are data reported from which it could be calculated? CR: percentage of participants cured (i.e. excreting no egg) at follow-up, among participants with confirmed infection (i.e. excreting eggs) at baseline. |
| **D4_ERR** | Dummy variable (**1** = Yes/**0** = No). Was the egg reduction rate (ERR) calculated at any time of follow-up? ERR: percentage reduction from (mean) baseline egg count, after treatment. Also referred to as “FECR” or Fecal Egg Count Reduction. Usually calculated using mean egg counts pre- and post- treatment, but occasionally calculated as mean individual reduction in egg count. If this is the method reported, this is noted. |
| **D4_intens** | Dummy variable (**1** = Yes/**0** = No). Was there a quantitative comparison of pre- and post-treatment intensity of infection? This variable is particularly relevant for studies which do not report the ERR, but still report and compare the intensity of infection before and after treatment as a mean, median and/or other quantitative measure, which would essentially enable the calculation of an ERR (i.e. not only qualifying the intensity as ‘low’ or ‘heavy’). |
| **D5_eggsAve** | Factor variable, describing the measure(s) of central tendency utilised either to calculate the ERR or to report the intensity (if D4_intens is 1): enter all relevant from: “**arithmetic**”; “**geometric**”; “**log-transformed**”; “**median**”, separated by semicolons. Enter “**unclear**” otherwise. |
| **D5_eggsMethod** | formula used for calculating (geometric) mean, if given. |
| **#efficacy Comment** | Free text: notes on how efficacy is calculated or reported |

# Participant characteristics

## 4.1 Soil-transmitted helminth parasites of interest: inclusion/exclusion criteria

| **E1_STHinclude** | Free text: other criteria related to soil-transmitted helminthiasis (STH) parasites of interest and that were applied to include participants – e.g. minimum number of eggs required. Enter “none” otherwise. |
| --- | --- |
| **E1_STHexclude** | Free text: other criteria related to STH parasites of interest and that were applied to exclude participants – e.g. symptomatic form of the disease. Enter “none” otherwise. |

## 4.2 What major co-infections were controlled for, and possibly resulted in exclusion from the study?

| **E2_malaria** | Dummy variable (**1** = Yes/**0** = No). Was co-infection with malaria assessed (at least at baseline) using a diagnostic test? |
| --- | --- |
| **E2_excluMal** | Dummy variable (**1** = Yes/**0** = No). Did infection with malaria (as confirmed by diagnostic test or clinical assessment only) result in exclusion from the study? |
| **E2_HIV** | Dummy variable (**1** = Yes/**0** = No). Was HIV status assessed, or knowledge thereof confirmed, (at least at baseline)? |
| **E2_excluHIV** | Dummy variable (**1** = Yes/**0** = No). Did known infection with HIV result in exclusion from the study? |
| **E2_incluHIV** | Dummy variable (**1** = Yes/**0** = No). Did known infection with HIV result in inclusion to the study? |
| **E3_helminth** | Was co-infection with at least 1 **other** helminth **(excluding soil-transmitted helminths of interest)** assessed (at least at baseline) using a diagnostic test? Use names of helminths separated by semicolons e.g. *Schistosoma mansoni*; *Taenia*; *Strongyloides stercoralis.* |
| **E3_helminthCom** | Free text: comments relating to entries in **E3_helminth** e.g. level of infection. |
| **E3_excluHelm** | Did infection with at least 1 **other** helminth **(excluding soil-transmitted helminths of interest)** result in exclusion from the study? Use names of helminths separated by semicolons. |
| **E3_incluHelm** | Did infection with at least 1 **other** helminth **(excluding soil-transmitted helminths of interest)** result in inclusion to the study? Use names of helminths separated by semicolons. |

## 4.3 Age range and pregnancy testing, inclusion or exclusion of pregnant participants

| **F0_ageMin (resp. Max) / F0_ageMax** | Minimum (resp. Maximum) age, in years, among enrolled participants. Strictly speaking, this does not necessarily correspond to the age range specified in inclusion/exclusion criteria for the study. Enter “**NA**” if not given. |
| --- | --- |
| **F1_ageCat** | SAC = School age children. Enter either "**pre-SAC**", "**SAC**", "**adult**", "**mothers**" or combinations separated by semicolons. “Mothers” is included as a category to help identify such cohorts which do not include all adults. |
| **F2_ageRangeEst** | use if age range can be estimated but not given for certain e.g. if there are a subset of school years tested or a mean and standard deviation of ages given. Age correspondence to school years is not always certain. Use free text as appropriate. |
| **F3_singleSex** | enter "**male**" or "**female**" if there is only one sex specifically included in the study. Otherwise leave blank. |
| **F5_pregnancy** | enter either "**no test indicated**", "**excluded**", "**included**", "**pregnant only**".  no assumptions about likely age for pregnancy to be a concern. |
| **F5_pregDefn** | free text indicating definition and/or test for pregnancy ("pregnancy" may include lactating). |

## 4.4 Specific inclusion criteria[[3]](#footnote-4)

| **G0_inclu Healthy** | Free text: general health status required for inclusion, along with criteria for judgement (e.g. “appear healthy at enrolment, as assessed by the study physician”, “no diarrhoea”). |
| --- | --- |
| **G1_incluMorph** | Semi-free text: weight and/or height limits for inclusion (e.g. “weight > 25 kg”). |
| **G3_inclu Compliant** | Free text: ability and willingness to follow/minimal compliance to the protocol required for inclusion, especially in analyses (e.g. “able and willing to be examined by the study physician at baseline and follow-up”). |
| **G4_inclu Consent** | Free text: method for securing informed consent, including who gave consent for children (parent, head of the school, etc.) and how (orally, in writing, etc.). |
| **G5_inclu School** | Free text: school grades among which children were recruited, and/or other criteria for inclusion related to the school (e.g. “be present at school on screening day” or “school accessible by road during rainy season”). |
| **G6_inclu Residency** | Free text: duration of residency in the study area, and/or other criteria of inclusion related to the area of residence (e.g. “living in the study area and intending to deliver at the Entebbe Hospital” or “residing in the study area since birth”). |
| **G7_incluOther** | Free text: other inclusion criteria not falling into any of the previous categories. |

## 4.5 Specific exclusion criteria^1^

| **H1_exclu Malnutrition** | Free text: state of malnutrition and/or anaemia leading to exclusion, along with decision rule if available (e.g. “signs of micronutrient deficiencies” or “signs of severe malnutrition (defined as children with weight/height ratio <3 SD or <70% of the median of WHO standardised reference values, or still with symmetrical oedema affecting both feet)”). |
| --- | --- |
| **H0_excluIllness** | Free text: list of severe illnesses or systematic diseases leading to exclusion. HIV and malaria are noted in specific columns elsewhere. |
| **H4_exclu Allergy** | Free text: precisions on allergies leading to exclusion, essentially hypersensitivity to study drugs. |
| **H5_exclu Medication** | Free text: medication(s) taken during a specific period prior to (or during) the study and leading to exclusion. **Often, any anthelmintic** within a defined prior time period. |
| **H7_exclu Participation** | Free text: multiple participation in the same or similar studies, or concomitant participation in another trial leading to exclusion. |
| **H8_excluOther** | Free text: other exclusion criteria not falling into any of the previous categories. |

# Per arm data: participants for drug efficacy estimation

| **X0_authors, X0_pubYear, X0_pubTitle,**  **X_ID** | Publication details and identifier assigned in elimination dataset. |
| --- | --- |
| **X_cohort** | Unique cohort identifier given as soon as a new cohort (set of participants) is added to the dataset, as assigned in cohorts dataset. |
| **X_arm** | Unique arm identifier given to each of the treatment arms in each cohort in the study. |

## 5.1 Number of participants: recruited, treated, and followed up within 60 days of treatment

| **J0_examined_wholeStudy** | If required for an estimation of participant numbers or gender balance, enter the number of participants examined for the whole study here. Entries in this column may correspond to a whole study or cohort rather than a row (arm). This entry is not essential (leave blank if not required) and the item recorded here will vary depending on the context. |
| --- | --- |
| **J0_assigned** | Total number of participants in the initial cohort, per arm;   - who were *included into the study and assigned to the intervention* (drug(s), control, placebo, no-treatment, etc.), with the intention to treat and follow them up for outcome assessment. Enter “NA” if unknown. |
| **J0_treated** | Total number of participants in the cohort, per arm;   - who were *included into the study and assigned to* ***and received*** *the intervention* (drug(s), control, placebo, no-treatment, etc.), with the intention to follow them up for outcome assessment. Enter “NA” if unknown |
| **J1_followed** | Total number of participants in the cohort, per arm;   - among **J0_treated** who were followed-up, at a pre-60 day follow up. If follow-up is detailed for multiple (pre-60 day) time-points, enter the maximum number of participants followed-up.   Enter “NA” if unknown. |
| **J1_incluDefn**  **ByFollowed** | Enter “yes” if ‘provide samples at follow-up’ is an inclusion criterion, meaning that the study cohort was defined *a posteriori*, based on compliance, and “no” if not. Enter “NA” if this is not clearly stated. |
| **J2_efficacy_n** | Number of participants whose individual data would enable estimation of drug efficacy against *Ascaris lumbricoides*, *Trichuris trichiura*, or hookworm, estimated if necessary.  participants who were:   - *diagnosed with at least 1 of the* 3 *soil-transmitted helminthiasis parasites of interest at baseline and* - *received an anthelmintic drug or comparator and* - *were followed up for outcome assessment within 60 days post-treatment.*   In some cases (especially, studies in which individuals non-infected with STH of interest at baseline were treated in the study), this exact number of participants is unknown. Estimate using the reported data. Detail method of estimation in J2_effEstMethod.  Enter “**NA**” if insufficient data for any estimation. |
| **J2_effEstimated** | Is the number in J2_efficacy_n an estimate? Yes/no. |
| **J2_estMethod** | Details on the method used for estimation of J2_efficacy_n. Enter all method details necessary, with any additional information such as reported prevalence numbers in **#armPartNotes.** For the major, most commonly used assumptions, enter as appropriate:  “**use baseline prevalence**” OR  “**use prevalence of most common (Al/Tt/HW)**” OR  “**use baseline prevalence of most common (Al/Tt/HW)**”  and, if also relevant:  “**assume same prevalence across groups**”  “**assume dropout** **proportional to arm size**”  “**assume reported are treated and followed**”.  Separate multiple aspects of method by “**;** “  See S3 Text for details of main assumptions used in estimation of J2_efficacy. |
| **F4_female** | Where a gender breakdown is reported, number of participants reported as female. |
| **F4_female_reportLevel** | Level, of J0_assigned / J0_treated / J1_followed / J2_efficacy_n or other descriptor, at which F4_female is reported. |
| **F4_female_denom** | Denominator relevant for estimating proportion of females in study arm. |
| **F4_female_prop** | Estimate of the proportion of females in the study, calculated from F4_female and F4_female_denom. |
| **#cohort** | Note on reason for any division into cohorts, to aid data extraction. |
| **K0_preTreatment** | Is the treatment given as a pre-treatment to the main aim of the study, usually in the case of a study investigating the effect of an (non-drug) intervention on reinfection? Yes/no. |
| **J3_infectOnly_treated** | Semi-free text indicating whether:   - all participants were treated regardless of infection status (enter “**no**”), or, - infected participants only were treated. Indicate what diagnosis resulted in treatment, entering the abbreviations: “**Al**” *A. lumbricoides*; “**Tt**”, *T. trichiura*; “**HW**”, hookworm; “**Ss**”, *Strongyloides stercoralis*. If multiple species apply, use AND or OR as appropriate. |
| **#armPartNotes** | any additional notes on the participants in this arm, particularly treatment criteria, any prevalences or other numbers required for estimation of J2_efficacy_n. |

# Drug treatment regimen for each arm

## 6.1 Details of drug regimen given to participants in each group

NB This schema fits all reported anthelmintic regimes of interest; in the small number of instances where other drugs are given in more complex regimes, for example 3 age categories, notes are made in #**treatmentnotes**

| **K0_armDesc** | Brief free text describing the cohort and arm in that line |
| --- | --- |
| **K1_drug1** | Name of first drug given as part of the assigned regimen. |
| **K1_drug1_doseByAge** | Was the dosage of drug 1 dependent on age? Yes/no. If yes, then columns including “dose1” indicate dosage given to the oldest age group. |
| **K1_drug1_dose1_dosePerDay** | Numerical entry only; enter amount of drug given per day, with the corresponding unit given in the next column (**K1_drug1_unit**) as reported. |
| **K1_drug1_doseUnit** | Unit corresponding to the amount of drug given per day, as reported. |
| **K1_drug1_dose1_splitPerDay** | Number of doses in one day that the daily amount is split into, if reported. |
| **K1_drug1_dose1_multiDays** | Was the daily dose given for multiple days? Yes/no. This variable is a double-check for multiple days treatment; if yes, then **K1_drug1_dose1_days** will be >1. |
| **K1_drug1_dose1_daysPerDose** | Days separating doses. Enter 1 if one dose given. |
| **K1_drug1_dose1_days** | Total number of days which this drug (at this dosage) was given on. |
| **K2_drug1_dose1_n** | For arms where dosage is dependent on age, the number of participants given dose1 (i.e. the number of participants in the oldest dosage group), if given. Enter NA if not given or if not relevant. |
| **K2_drug1_ageSplit** | For arms where dosage is dependent on age, age in years of youngest participants in older dosage group. |
| **[K2 …rows including “dose2”]** | As per corresponding columns for dose1, the details for the dose regime given to the lower age category. Enter NA if not relevant. |
| **K3_drug2** | Name of second drug given in a combination. Enter “**none**” if a single drug is given to this arm. |
| **K3 rows for “drug2”** | As per corresponding columns for drug1, if there is a combination of drugs administered to participants. Leave blank if K3_drug2 = “**none**”. |
|  |  |
| **K4_daysBetweenMultiDrugs** | Number of days between administering drug 1 and drug 2 in the combination. Enter NA if not given, leave blank if K3_drug2 = “**none**”. |
| **K5_addPZQ** | Could praziquantel (PZQ) have been given to any participants in this arm, either routinely or if a relevant infection was diagnosed? Enter “**yes**” if PZQ was reported as part of the potential treatment for any participants in this arm, clarify if necessary (e.g. different doses for different parasites) leave blank otherwise. |
| **K6_addDrug** | Could any other drug (not including any anthelmintics, which should be part of the treatment details) have been administered to any participants in this arm. Enter name of drug(s). |
| **#treatmentNotes** | Any notes on treatments given in this arm. |

1. Keiser J, Utzinger J. Efficacy of current drugs against soil-transmitted helminth infections: systematic review and meta-analysis. JAMA. 2008;299: 1937-1948. doi: 10.1001/jama.299.16.1937. [↑](#footnote-ref-2)
2. Julé AM, Vaillant M, Lang TA, Guérin PJ, Olliaro PL. The schistosomiasis clinical trials landscape: a systematic review of antischistosomal treatment efficacy studies and a case for sharing individual participant-level data (IPD). PLoS Negl Trop Dis. 2016;10: e0004784. doi:10.1371/journal.pntd.0004784. [↑](#footnote-ref-3)
3. When inconsistency between sources (e.g. trial registry and journal article), report most stringent criteria; unless additional information permits to say which source is accurate. [↑](#footnote-ref-4)
