# Supplementary material for: Systematic review of studies generating individual participant data on the efficacy of drugs for treating soil-transmitted helminthiases and the case for data-sharing
Source: PLoS Negl Trop Dis. 2017 Oct 31;11(10):e0006053. doi: 10.1371/journal.pntd.0006053 (PMC5681297; doi:10.1371/journal.pntd.0006053)
Supplement: S3 Text — (DOCX) [file pntd.0006053.s003.docx]

S3 Text Estimating number of individual participant data

We estimated the number of relevant individual participant data (IPD) where it was not clear how many participants’ data could be used for an estimate of drug efficacy; i.e. the number of treated participants, positively diagnosed at baseline, and followed up 60 days or less after treatment. We adapted our estimation procedure depending on the reported items in the publication. Calculations in addition to those listed below are clarified in the column [J2_effEstMethod] of S3 Dataset and may include use of numbers of participants involved in other parts of the study.

The principal estimation methods common to multiple studies are described as follows. Abbreviations used throughout: STH, soil-transmitted helminthiasis; Al, *Ascaris lumbricoides*; Tt, *Trichuris trichiura*; HW, hookworm.

1. If a baseline (pre-treatment) prevalence of the three soil-transmitted helminth infections of interest (combined) was reported, and there is loss to follow-up, this was multiplied by the number of participants followed up, making the assumption that the loss to follow up was not dependent on infection status. This method is referred to as “**use baseline prevalence**” in S3 Dataset column [J2_effEstMethod].
2. If infection prevalence was reported individually, but with no clarification of the prevalence of single or multiple species, then the most common infection is the lower bound of the combined prevalence, since most conservatively, all other infections could be concomitant with the most common. This lower bound was taken as the estimate of STH prevalence (before or after treatment as reported). This method is referred to as “**use prevalence of most common (Al/Tt/HW)**” (using the appropriate parasite in parentheses) in S3 Dataset column [J2_effEstMethod].
3. If both 1 and 2 apply, refer to this as “**use baseline prevalence of most common (Al/Tt/HW)**” (using the appropriate parasite in parentheses).
4. If prevalence of STH (or individual infection prevalence) is reported across arms/cohorts, we assumed that prevalence did not significantly differ across arms/cohorts. This method is referred to as “**assume same (Al/Tt/HW) prevalence across groups**” (with infection clarification as appropriate) in S3 Dataset column [J2_effEstMethod].
5. If total loss to follow-up across all treatment arms was reported, but not broken down by arm, loss to follow up was calculated as proportional to the size of arms (assuming no biased loss across arms). This method is referred to as “**assume dropout** **proportional to arm size**” in S3 Dataset column [J2_effEstMethod].
6. If no loss to follow-up was mentioned, we assumed that the numbers reported are those who were treated and followed up successfully. This method is referred to as “**assume reported are treated and followed**” in S3 Dataset column [J2_effEstMethod].
7. For the small number of arms with very little information reported, no estimate was made. This is referred to by an “**NA**” in S3 Dataset column [J2_efficacy_n].
