# Supplementary figures and images for: Systematic review of studies generating individual participant data on the efficacy of drugs for treating soil-transmitted helminthiases and the case for data-sharing
Source: PLoS Negl Trop Dis. 2017 Oct 31;11(10):e0006053. doi: 10.1371/journal.pntd.0006053 (PMC5681297; doi:10.1371/journal.pntd.0006053)

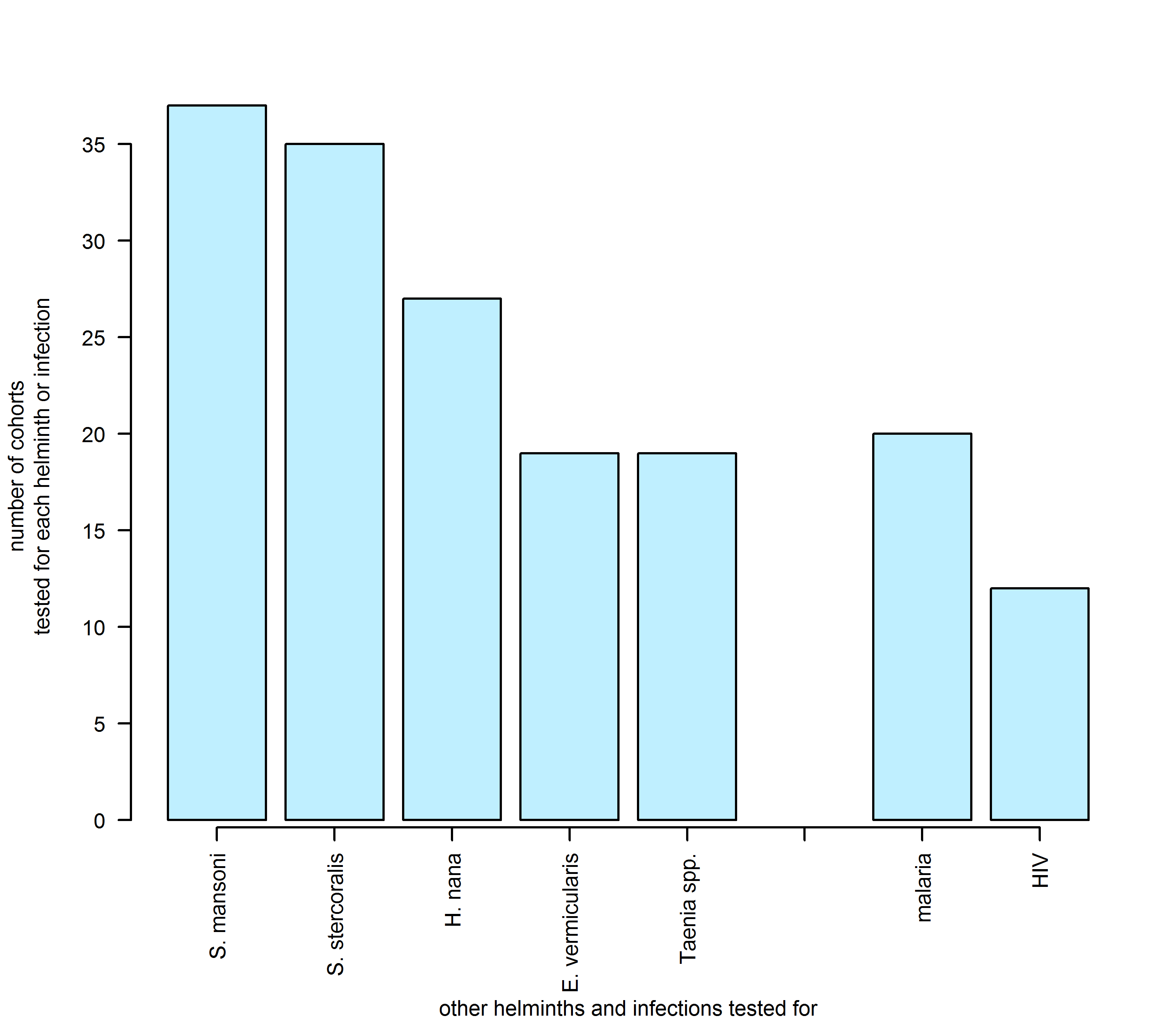

Supplement: S1 Fig — Abbreviations from left to right: S. mansoni, Schistosoma mansoni; S. stercoralis, Strongyloides stercoralis; H. nana, Hymenolepis nana; E. vermicularis, Enterobius vermicularis; Taenia spp., Taenia species; HIV, human immunodeficieny virus. (TIF) [file pntd.0006053.s005.tif]
